# Recombinant human C1 esterase inhibitor (conestat alfa) in the prevention of severe SARS-CoV-2 infection in hospitalized patients with COVID-19: A structured summary of a study protocol for a randomized, parallel-group, open-label, multi-center pilot trial (PROTECT-COVID-19)

**DOI:** 10.1186/s13063-020-04976-x

**Published:** 2021-01-04

**Authors:** Pascal Urwyler, Panteleimon Charitos, Stephan Moser, Ingmar A. F. M. Heijnen, Marten Trendelenburg, Reto Thoma, Johannes Sumer, Adrián Camacho-Ortiz, Marcelo R. Bacci, Lars C. Huber, Melina Stüssi-Helbling, Werner C. Albrich, Parham Sendi, Michael Osthoff

**Affiliations:** 1 Division of Internal Medicine, University Hospital Basel, Basel, Switzerland; 2 Division of Medical Immunology, Laboratory Medicine, University Hospital Basel, Basel, Switzerland; 3 Department of Clinical Research and Department of Biomedicine, University of Basel, Basel, Switzerland; 4 Division of Infectious Diseases and Hospital Epidemiology, Cantonal Hospital St. Gallen, St. Gallen, Switzerland; 5 Hospital Universitario Dr. José Eleuterio González, Facultad de Medicina, Universidad Autónoma de Nuevo León, Monterrey, Mexico; 6 Department of General Practice, Centro Universitário em Saúde do ABC, Santo André, SP Brazil; 7 Clinic for Internal Medicine, City Hospital Triemli, Zurich, Switzerland; 8 Department of Infectious Diseases and Hospital Epidemiology, University Hospital Basel, Basel, Switzerland; 9 Institute for Infectious Diseases, University of Bern, Bern, Switzerland

**Keywords:** COVID-19, randomized trial, protocol, C1 esterase inhibitor, complement system, kallikrein kinin system, contact activation system

## Abstract

**Objectives:**

Conestat alfa, a recombinant human C1 esterase inhibitor, is a multi-target inhibitor of inflammatory cascades including the complement, the kinin-kallikrein and the contact activation system. The study objective is to investigate the efficacy and safety of conestat alfa in improving disease severity and short-term outcome in COVID-19 patients with pulmonary disease.

**Trial design:**

This study is an investigator-initiated, randomized (2:1 ratio), open-label, parallel-group, controlled, multi-center, phase 2a clinical trial.

**Participants:**

This trial is conducted in 3 hospitals in Switzerland, 1 hospital in Brazil and 1 hospital in Mexico (academic and non-academic). All patients with confirmed SARS-CoV-2 infection requiring hospitalization for at least 3 calendar days for severe COVID-19 will be screened for study eligibility.

Inclusion criteria:

- Signed informed consent

- Age 18-85 years

- Evidence of pulmonary involvement on CT scan or X-ray of the chest

- Duration of symptoms associated with COVID-19 ≤ 10 days

- At least one of the following risk factors for progression to mechanical ventilation on the day of enrolment:

1) Arterial hypertension

2) ≥ 50 years

3) Obesity (BMI ≥ 30 kg/m2)

4) History of cardiovascular disease

5) Chronic pulmonary disease

6) Chronic renal disease

7) C-reactive protein > 35mg/L

8) Oxygen saturation at rest of ≤ 94% when breathing ambient air

Exclusion criteria:

- Incapacity or inability to provide informed consent

- Contraindications to the class of drugs under investigation (C1 esterase inhibitor)

- Treatment with tocilizumab or another IL-6R or IL-6 inhibitor before enrolment

- History or suspicion of allergy to rabbits

- Pregnancy or breast feeding

- Active or anticipated treatment with any other complement inhibitor

- Liver cirrhosis (any Child-Pugh score)

- Admission to an ICU on the day or anticipated within the next 24 hours of enrolment

- Invasive or non-invasive ventilation

- Participation in another study with any investigational drug within the 30 days prior to enrolment

- Enrolment of the study investigators, their family members, employees and other closely related or dependent persons

**Intervention and comparator:**

Patients randomized to the experimental arm will receive conestat alfa in addition to standard of care (SOC). Conestat alfa (8400 U followed by 4200 U every 8 hours) will be administered as a slow intravenous injection (5-10 minutes) over a 72-hour period (i.e. 9 administrations in total). The first conestat alfa treatment will be administered on the day of enrolment. The control group will receive SOC only. SOC treatment will be administered according to local institutional guidelines, including supplemental oxygen, antibiotics, corticosteroids, remdesivir, and anticoagulation.

**Main outcomes:**

The primary endpoint of this trial is disease severity on day 7 after enrolment assessed by an adapted WHO Ordinal Scale for Clinical Improvement (score 0 will be omitted and score 6 and 7 will be combined) from 1 (no limitation of activities) to 7 (death).

Secondary outcomes include (i) the time to clinical improvement (time from randomization to an improvement of two points on the WHO ordinal scale or discharge from hospital) within 14 days after enrolment, (ii) the proportion of participants alive and not having required invasive or non-invasive ventilation at 14 days after enrolment and (iii) the proportion of subjects without an acute lung injury (defined by PaO_2_/FiO_2_ ratio of ≤300mmHg) within 14 days after enrolment.

Exploratory outcomes include virological clearance, C1 esterase inhibitor pharmacokinetics and changes in routine laboratory parameters and inflammatory proteins.

**Randomisation:**

Subjects will be randomised in a 2:1 ratio to treatment with conestat alfa in addition to SOC or SOC only. Randomization is performed via an interactive web response system (SecuTrial®).

**Blinding (masking):**

In this open-label trial, participants, caregivers and outcome assessors are not blinded to group assignment.

**Numbers to be randomised (sample size):**

We will randomise approximately 120 individuals (80 in the active treatment arm, 40 in the SOC group). Two interim analyses after 40 and 80 patients are planned according to the Pocock adjusted levels α_p_ = 0.0221. The results of the interim analysis will allow adjustment of the sample size (Lehmacher, Wassmer, 1999).

**Trial Status:**

PROTECT-COVID-19 protocol version 3.0 (July 07 2020). Participant recruitment started on July 30 2020 in one center (Basel, Switzerland, first participant included on August 06 2020). In four of five study centers patients are actively recruited. Participation of the fifth study center (Mexico) is anticipated by mid December 2020. Completion of trial recruitment depends on the development of the SARS-CoV-2 pandemic.

**Trial registration:**

Clinicaltrials.gov, number: NCT04414631, registered on 4 June 2020

**Full protocol:**

The full protocol is attached as an additional file, accessible from the Trials website (Additional file 1). In the interest of expediting dissemination of this material, the familiar formatting has been eliminated; this Letter serves as a summary of the key elements of the full protocol.

**Supplementary Information:**

The online version contains supplementary material available at 10.1186/s13063-020-04976-x.

## Supplementary Information

**Additional file 1.** Full Study Protocol.

## Data Availability

All co-authors will have access to the original dataset. The data will be available from the author on reasonable request by email.

