# Supplementary material for: Recombinant human C1 esterase inhibitor (conestat alfa) in the prevention of severe SARS-CoV-2 infection in hospitalized patients with COVID-19: A structured summary of a study protocol for a randomized, parallel-group, open-label, multi-center pilot trial (PROTECT-COVID-19)
Source: Trials. 2021 Jan 4;22:1. doi: 10.1186/s13063-020-04976-x (PMC7780206; doi:10.1186/s13063-020-04976-x)
Supplement: Supplementary file 1 — Additional file 1. Full Study Protocol. [file 13063_2020_4976_MOESM1_ESM.pdf]

Clinical Study Protocol

Recombinant human C1 esterase inhibitor (conestat alfa) in the prevention of severe SARS-CoV-2 infection in hospitalized patients with COVID-19: a randomized, parallel-group, open-label, multi-center pilot trial (PROTECT-COVID-19).

Short title: PROTECT-COVID-19: Randomisierte, unverblindete, kontrollierte Pilotstudie zur Beurteilung der Wirksamkeit und Sicherheit einer Gabe von Conestat alfa in Bezug auf die Progression einer Infektion mit SARS-CoV-2 bei hospitalisierten Patienten

Study Type:	Investigator initiated clinical trial with Investigational Medicinal Product (IMP)
Study Categorisation:	Risk category C
Study Registration:	EudraCT number: 2020-002520-36<br>Clinicaltrials.gov number: NCT04414631<br>Swiss National Clinical Trials Portal (SNCTP) at www.kofam.ch: SNCTP000003972
Study Identifier:	PROTECT-COVID-19
Sponsor, Sponsor-Investigator or Principal Investigator:	PD Dr. med. Michael Osthoff<br>University Hospital Basel, Division of Internal Medicine, Petersgraben 4, CH-4031 Basel<br>Tel.: +41 61 328 6828<br>Fax.: +41 61 265<br>Email: michael.osthoff@usb.ch
Investigational Product:	Conestat alfa (Ruconest®)
Protocol Version and Date:	Version 3.0, 07.07.2020

CONFIDENTIAL

The information contained in this document is confidential and the property of the “sponsor”. The information may not - in full or in part - be transmitted, reproduced, published, or disclosed to others than the applicable Competent Ethics Committee(s) and Regulatory Authority(ies) without prior written authorisation from the sponsor except to the extent necessary to obtain informed consent from those who will participate in the study.

Signature Page(s)

Study number	EKNZ 2020-01252
Study Title	Recombinant human C1 esterase inhibitor (conestat alfa) in the prevention of severe SARS-CoV-2 infection in hospitalized patients with COVID-19: a randomized, parallel-group, open-label, multi-center pilot trial (PROTECT-COVID-19).

The Sponsor-Investigator and trial statistician have approved the protocol version 3.0 dated 07.07.2020, and confirm hereby to conduct the study according to the protocol, current version of the World Medical Association Declaration of Helsinki, ICH-GCP guidelines or ISO 14155 norm if applicable and the local legally applicable requirements.

Sponsor-Investigator:

Place/Date Signature

Sub-Investigator: Prof. Dr. med. Marten Trendelenburg

Place/Date Signature

Sub-Investigator: Prof. Dr. med. Parham Sendi

Place/Date Signature

Signature Page(s)

Study number	EKNZ 2020-01252
Study Title	Recombinant human C1 esterase inhibitor (conestat alfa) in the prevention of severe SARS-CoV-2 infection in hospitalized patients with COVID-19: a randomized, parallel-group, open-label, multi-center pilot trial (PROTECT-COVID-19).

Local coordinating Investigator/Sub-Investigator at study site*:

I have read and understood this trial protocol and agree to conduct the trial as set out in this study protocol, the current version of the World Medical Association Declaration of Helsinki, ICH-GCP guidelines or ISO 14155 norm and the local legally applicable requirements.

Site (name and address):

____

____

____

Locally coordinating Investigator/Sub-Investigator (printed name):

____

____________________	__________________________
Place/Date	Signature

Table of Contents

STUDY SYNOPSIS **7**
ABBREVIATIONS **12**
STUDY SCHEDULE **13**
1. STUDY ADMINISTRATIVE STRUCTURE **15**
1.1 Sponsor, Sponsor-Investigator 15
1.2 Principal Investigator(s) 15
1.3 Statistician ("Biostatistician") 15
1.4 Laboratory 15
1.5 Monitoring institution 15
1.6 Data Safety Monitoring Committee 15
1.7 Any other relevant Committee, Person, Organisation, Institution 16
2. ETHICAL AND REGULATORY ASPECTS **17**
2.1 Study registration 17
2.2 Categorisation of study 17
2.3 Competent Ethics Committee (CEC) 17
2.4 Competent Authorities (CA) 17
2.5 Ethical Conduct of the Study 18
2.6 Declaration of interest 18
2.7 Patient Information and Informed Consent 18
2.8 Participant privacy and confidentiality 19
2.9 Early termination of the study 19
2.10 Protocol amendments 19
3. BACKGROUND AND RATIONALE **20**
3.1 Background and Rationale 20
3.2 Investigational Product (treatment, device) and Indication 23
3.3 Preclinical Evidence 23
3.4 Clinical Evidence to Date 23
3.5 Dose Rationale / Medical Device: Rationale for the intended purpose in study (pre-market MD) 24
3.6 Explanation for choice of comparator (or placebo) 26
3.7 Risks / Benefits 26
3.8 Justification of choice of study population 27
4. STUDY OBJECTIVES **28**
4.1 Overall Objective 28
4.2 Primary Objective 28
4.3 Secondary Objectives 28
4.4 Safety Objectives 28
5. STUDY OUTCOMES **29**
5.1 Primary Outcome 29
5.2 Secondary Outcomes 29
5.3 Other Outcomes of Interest 30
5.4 Safety Outcomes 30
6. STUDY DESIGN **31**
6.1 General study design and justification of design 31

6.2 Methods of minimising bias 31
6.2.1 Randomisation 31
6.2.2 Blinding procedures 31
6.2.3 Other methods of minimising bias 31
6.3 Unblinding Procedures (Code break) 31
7. STUDY POPULATION 32
7.1 Eligibility criteria 32
7.2 Recruitment and screening 33
7.3 Assignment to study groups 33
7.4 Criteria for withdrawal / discontinuation of participants 33
8. STUDY INTERVENTION 34
8.1 Identity of Investigational Products (treatment / medical device) 34
8.1.1 Experimental Intervention (treatment) 34
8.1.2 Control Intervention (standard/routine/comparator treatment / medical device) 34
8.1.3 Packaging, Labelling and Supply (re-supply) 34
8.1.4 Storage Conditions 34
8.2 Administration of experimental and control interventions 34
8.2.1 Experimental Intervention 34
8.2.2 Control Intervention 35
8.3 Dose / Device modifications 35
8.4 Compliance with study intervention 35
8.5 Data Collection and Follow-up for withdrawn participants 35
8.6 Trial specific preventive measures 35
8.7 Concomitant Interventions (treatments) 36
8.8 Study Drug / Medical Device Accountability 36
8.9 Return or Destruction of Study Drug / Medical Device 36
9. STUDY ASSESSMENTS 37
9.1 Study flow chart(s) / table of study procedures and assessments 37
9.2 Assessments of outcomes 38
9.2.1 Assessment of primary outcome 38
9.2.2 Assessment of secondary outcomes 39
9.2.3 Assessment of other outcomes of interest 39
9.2.4 Assessment of safety outcomes 40
9.2.5 Assessments in participants who prematurely stop the study 40
9.3 Procedures at each visit 41
9.3.1 Split into subtitles by type of visit 41
10. SAFETY 43
10.1 Drug studies 43
10.1.1 Definition and assessment of (serious) adverse events and other safety related events 43
10.1.2 Reporting of serious adverse events (SAE) and other safety related events 44
10.1.3 Follow up of (Serious) Adverse Events 45
11. STATISTICAL METHODS 46
11.1 Hypothesis 46
11.2 Determination of Sample Size 46

11.3 Statistical criteria of termination of trial 46
11.4 Planned Analyses 46
11.4.1 Datasets to be analysed, analysis populations 46
11.4.2 Primary Analysis 47
11.4.3 Secondary Analyses 47
11.4.4 Interim analyses 47
11.4.5 Safety analysis 47
11.4.6 Deviation(s) from the original statistical plan 48
11.5 Handling of missing data and drop-outs 48
12. QUALITY ASSURANCE AND CONTROL 49
12.1 Data handling and record keeping / archiving 49
12.1.1 Case Report Forms 49
12.1.2 Specification of source documents 49
12.1.3 Record keeping / archiving 49
12.2 Data management 49
12.2.1 Data Management System 49
12.2.2 Data security, access and back-up 49
12.2.3 Analysis and archiving 50
12.2.4 Electronic and central data validation 50
12.3 Monitoring 50
12.4 Audits and Inspections 50
12.5 Confidentiality, Data Protection 50
12.6 Storage of biological material and related health data 51
13. PUBLICATION AND DISSEMINATION POLICY 52
14. FUNDING AND SUPPORT 53
14.1 Funding 53
14.2 Other Support 53
15. INSURANCE 53
16. REFERENCES 54
17. APPENDICES .... FEHLER! TEXTMARKE NICHT DEFINIERT.

STUDY SYNOPSIS

**Sponsor / Sponsor-Investigator**	PD Dr. med. Michael Osthoff
**Study Title:**	Recombinant human C1 esterase inhibitor (conestat alfa) in the prevention of severe SARS-CoV-2 infection in hospitalized patients with COVID-19: a randomized, parallel-group, open-label, multi-center pilot trial (PROTECT-COVID-19).
**Short Title / Study ID:**	PROTECT-COVID-19: conestat alfa in the prevention of SARS-CoV-2 infection related deterioration
**Protocol Version and Date:**	3.0, 07.07.2020
**Trial registration:**	EudraCT number: 2020-002520-36<br>Clinicaltrials.gov number: NCT04414631<br>Swiss National Clinical Trials Portal (SNCTP) at www.kofam.ch: SNCTP000003972
**Study category and Rationale**	Clinical trial of medicinal product, category C investigating an IMP that is not authorised in Switzerland.
**Clinical Phase:**	Phase 2a (therapeutic exploratory) trial

**Background and Rationale:**	Severe acute respiratory syndrome coronavirus 2 (SARS-CoV-2) has spread globally since December 2019 causing a pandemic of Coronavirus disease 19 (COVID-19) in almost all countries worldwide. The clinical spectrum of COVID-19 ranges from asymptomatic carriers to respiratory failure requiring respiratory support in the intensive care unit (ICU). Systemic hyperinflammation is a hallmark of more severe stages of COVID-19 leading to acute respiratory distress syndrome, mechanical ventilation and ultimately death. In this stage, COVID-19 is associated with a decrease in suppressor and regulatory T cell counts and an extensive release of proinflammatory cytokines and biomarkers called a cytokine storm, which is thought to be the major driver of severe pneumonia caused by SARS-CoV-2.<br><br>No proven or approved effective treatment for COVID-19 infection currently exists. The mechanism responsible for virus-induced hyper-activation of the host immune system remains poorly understood but likely involves several immune cells and inflammatory plasmatic cascades such as the complement and the kinin-kallikrein (KK) system. The complement system (CS) is an integral part of the innate immune system and consists of a number of distinct plasma proteins that act as a first line of defence inducing an inflammatory response after opsonisation of pathogens and dying cells. Previous evidence indicated that an over-activated complement systems, driven by the lectin pathway of complement in particular, seems to contribute to ALI in response to infection with CoV such as SARS-CoV-2 leading to the clinical picture of severe COVID-19 pneumonia and consequently to ARDS.<br><br>The KK system is a plasmatic cascade that after activation (shear stress of vessels, e.g. during vascular inflammation) and subsequent cleavage of kininogen by kallikrein releases bradykinin. Bradykinin binds to B2-receptors on endothelial cells leading to capillary leakage and angioedema. After enzymatic degradation bradykinin products may also bind to B1-receptors on endothelial cells that are upregulated under proinflammatory conditions and have strong vasopermeable capacity. Although direct evidence is lacking, several facts argue for an involvement of bradykinin in pulmonary angioedema observed in COVID-19, in particular a reduced activity of ACE2 caused by SARS-CoV-2 leading to a relative abundance of bradykinin degradation production with subsequent B1 activation and local pulmonary edema.<br><br>C1 esterase inhibitor (C1INH) is a member of the serpin superfamily of serine-protease inhibitors and is a strong inhibitor of the CS and KK system among others. Conestat alfa is a recombinant human C1INH, that shares an identical protein structure with plasma-derived C1INH. Although data on C1INH treatment in the context of SARS-CoV-2 infection are lacking, results from previous studies suggest that C1INH treatment may reduce the collateral damage caused by hyperinflammation in human sepsis. The rationale of the current trial is based upon the following assumptions: In the context of COVID-19, conestat alfa treatment may 1) dampen uncontrolled complement activation and collateral lung damage and 2) reduce capillary leakage and subsequent pulmonary edema by direct inhibition of KK system.<br><br>Hypothesis: Administration of conestat alfa for 72 hours in addition to standard of care (SOC) in patients hospitalized with non-critical SARS-CoV-2 pneumonia (WHO Ordinal Scale Score 3 or 4) will be associated with a reduced clinical severity on day 7 after inclusion and a lower risk of disease progression to ALI and ARDS.
**Objective(s):**	The primary objective of the study is to determine if adding 72 hours of treatment with conestat alfa to SOC treatment in adult participants admitted with non-critically ill COVID-19 will affect disease severity within 7 days after enrolment as assessed by the WHO Ordinal Scale for Clinical Improvement.<br><br>Secondary objectives will determine if conestat alfa will<br>- Reduce the time to clinical improvement (time from randomisation to an improvement of two points on the WHO ordinal scale or live discharge from hospital, whichever came first) within 14 days after enrolment.<br>- Increase the proportion of participants alive and not having required invasive or non-invasive ventilation at 14 days after enrolment.<br>- Reduce the proportion of subjects with an ALI (defined by PaO_2/FiO_2 ratio of $\leq$300mmHg) within 14 days after enrolment

**Outcome(s):**	The primary endpoint will be the disease severity on the 7-point Ordinal WHO scale on day 7 (for the current study, score 0 will be omitted and score 6 and 7 will be combined). The ordinal scale measures illness severity over time.<br>Secondary endpoints include:<br>- Time to clinical improvement (time from randomisation to an improvement of two points on the seven-category WHO ordinal scale or live discharge from hospital, whichever came first) within 14 days after enrolment.<br>- Proportion of participants alive and not having required invasive or non-invasive ventilation at 14 days after enrolment.<br>- Proportion of subjects with an ALI (defined by PaO_2/FiO_2 ratio of ≤300mmHg) within 14 days after enrolment
**Study design:**	Randomized, open-label, parallel-group, controlled, multi-center clinical trial
**Inclusion / Exclusion criteria:**	Patients admitted for the management of confirmed COVID-19 will be approached.<br>Inclusion criteria: Male or female of age 18-85 years, admitted to the hospital because of confirmed (by a positive SARS-CoV-2 PCR result) COVID-19 infection, evidence of pulmonary involvement on CT scan or X-ray of the chest, symptom onset within the previous 10 days AND at least one additional risk factor for progression to mechanical ventilation: 1) arterial hypertension, 2) ≥50 years, 3) obesity (BMI≥30.0 kg/m2), 4) history of cardiovascular disease, 5) chronic pulmonary disease, 6) chronic renal disease, 7) C-reactive protein of >35mg/L, 8) oxygen saturation at rest in ambient air of ≤94%.<br><br>Exclusion criteria: Contraindications to the class of drugs under study (C1 esterase inhibitor), treatment with tocilizumab or another Il-6R or Il-6 inhibitor before enrolment, History or suspicion of allergy to rabbits, pregnancy or breast feeding, active or planned treatment with any other complement inhibitor, liver cirrhosis (any Child-Pugh score), currently admitted to an ICU or expected admission within the next 24 hours, currently receiving invasive or non-invasive ventilation (with the exception of high-flow oxygen therapy), participation in another study with investigational drug within the 30 days preceding (with the exception of other COVID-19 studies)

<table>
<tr><td>Measurements and procedures:</td><td>After providing informed consent a pregnancy test will be performed if appropriate. Only subject who fulfilling all eligibility criteria will be randomised in a 2:1 ratio in an open-label controlled design to treatment with conestat alfa in addition to SOC or SOC only starting on day 0. The first conestat alfa treatment will usually be administered on the same day and continued for a maximum of 64 hours (+/-4 hours).

Vital signs, disease severity, clinical improvement, admission to ICU, receipt of additional anti-inflammatory therapies such as tocilizumab and requirement for non-invasive or invasive ventilation will be documented. Disease severity will be evaluated by the WHO Ordinal Scale:
<table>
<tr><th>Patient State</th><th>Description</th><th>Score</th></tr>
<tr><td rowspan="2">Outpatient</td><td>No limitation in activities</td><td>1</td></tr>
<tr><td>Limitation in activities</td><td>2</td></tr>
<tr><td rowspan="2">Hospitalized
Mild disease</td><td>No oxygen therapy</td><td>3</td></tr>
<tr><td>Oxygen by mask or nasal prongs</td><td>4</td></tr>
<tr><td rowspan="2">Hospitalized
Severe disease</td><td>Non-invasive ventilation or high-flow oxygen</td><td>5</td></tr>
<tr><td>Intubation, mechanical ventilation +/- additional organ support</td><td>6</td></tr>
<tr><td>Death</td><td>Death</td><td>7</td></tr>
</table>
Virological clearance will be assessed at 14 days after enrolment or discharge. Routine laboratory parameters and changes in certain biomarker levels will be assessed during 14 days or until discharge.

C1INH pharmacokinetics and complement and inflammatory proteins will be assessed (in Basel, optional for other centers) during 14 days or until discharge.

Follow-up will include a structured telephone interview or study visit (if still admitted) after 4 weeks to assess adverse events and outcome.</td></tr>
<tr><td>Study Product / Intervention:</td><td>Conestat alfa (8400 U followed by 4200 U every 8 hours, 9 administrations in total) will be administered as a slow intravenous injection (5-10 minutes) over a 72 hour period. Both trial arms will receive SOC treatment according to local guidelines.</td></tr>
<tr><td>Control Intervention (if applicable):</td><td>Standard of care treatment including supplemental oxygen, antibiotics, off-label drugs for the treatment of COVID-19, and treatment for comorbid conditions and underlying disease.</td></tr>
<tr><td>Number of Participants with Rationale:</td><td>Approximately 120 subjects (80 in the active treatment arm, 40 in the SOC group).

The primary endpoint is a 7-point scale and the standard deviation σ can be expected as 1.5 points. A relevant effect δ is an advantage of at least 1 point. Then, the standardized difference is about $\delta/\sigma = 0.67$. For a fixed sample size design with a two-sided significance level of $\alpha = 0.05$ and a power of $1 - \beta = 0.80$, a sample size of N = 2 x 38 is necessary. For a 2:1-randomization, a nonparametric analysis by the stratified logrank-test, and an adaptive group sequential analysis, the overall sample size is estimated as 120 = 80 + 40.

Two interim analyses after 40 and 80 patients are planned according to the Pocock adjusted levels $\alpha_p = 0.0221$. Based on the results of an interim analysis the sample size can be adjusted (Lehmacher, Wassmer, 1999). There are no prespecified futility margins, but the DSMB can stop the study in the case of insufficient interim results.</td></tr>
<tr><td>Study Duration:</td><td>12 months</td></tr>
<tr><td>Study Schedule:</td><td>7/2020 of First-Participant-In (planned)

6/2021 of Last-Participant-Out (planned)</td></tr>
</table>

**Investigator(s):**	PD Dr. med. Michael Osthoff, University Hospital Basel, Division of Internal Medicine, Petersgraben 4, 4031 Basel, Tel. +41 61 328 6828. Fax. +41 61 265 4722, e-mail michael.osthoff@usb.ch
**Study Centre(s):**	Multi-national study (it is planned to recruit patients in approximately 4-6 centers in Switzerland, Brazil and Mexico)
**Statistical Considerations:**	Detailed methodology for statistical analyses of the data collected in this trial will be documented in a Statistical Analysis Plan.<br>Full Analysis Set/Intent-to-Treat Population: The FAS/ITT Population is defined as all patients who are randomly allocated to a study arm. Statistical analyses will be based on the treatment arm to which the patient was allocated.<br>Safety Population: The Safety Population is defined as all patients who received at least one dose of conestat alfa.<br>The primary endpoint WHO 7-point outcome scale at day 7 will be analyzed by nonparametric logrank test stratified by its baseline values with two-sided α-level of 5 %.<br>The secondary endpoint is time to improvement of at least 2 points. It will be tested only after a significant test of the primary endpoint (a priori ordered hypotheses), therefore, no alpha adjustment is necessary.<br>Furthermore, 95% confidence intervals will be determined, and the results will be presented graphically by means of box-and-whisker plots.<br>Time to event (e.g. death, virological clearance, defervescence) will be displayed by Kaplan-Meier plots and compared with a logrank test. Appropriate statistical tests will be used for the analysis of other outcomes of interest and will be detailed in the statistical analysis plan prior to unlocking of the study database.
**GCP Statement:**	This study will be conducted in compliance with the protocol, the current version of the Declaration of Helsinki, the ICH-GCP or ISO EN 14155 (as far as applicable) as well as all national legal and regulatory requirements.

ABBREVIATIONS

Provide a list of abbreviations used on the protocol - to be completed

AE	Adverse Event
BASEC	Business Administration System for Ethical Committees, (https://submissions.swissethics.ch/en/)
CA	Competent Authority (e.g. Swissmedic)
CEC	Competent Ethics Committee
CS	Complement system
C1INH	C1 esterase inhibitor
COVID-19	Coronavirus disease 2019
CRF	Case Report Form
ClinO	Ordinance on Clinical Trials in Human Research *(in German: KlinV, in French: OClin, in Italian: OSRUm)*
eCRF	Electronic Case Report Form
CTCAE	Common terminology criteria for adverse events
DSUR	Development safety update report
GCP	Good Clinical Practice
IB	Investigator's Brochure
Ho	Null hypothesis
H1	Alternative hypothesis
HRA	Federal Act on Research involving Human Beings *(in German: HFG, in French: LRH, in Italian: LRUm)*
IMP	Investigational Medicinal Product
IIT	Investigator-initiated Trial
ISO	International Organisation for Standardisation
ITT	Intention to treat
KK	Kinin-kallikrein
MD	Medical Device
MedDO	Medical Device Ordinance *(in German: MepV, in French: ODim)*
PI	Principal Investigator
SARS-CoV-2	Severe acute respiratory syndrome-coronavirus-2
SDV	Source Data Verification
SOP	Standard Operating Procedure
SPC	Summary of product characteristics
SUSAR	Suspected Unexpected Serious Adverse Reaction
TMF	Trial Master File

STUDY SCHEDULE

Study Periods	Screening	Intervention Period				Follow-up	
Visit	1	2	3	4	5	6	7
Time (weeks or days, or hours)	d-1 to d0[1]	d0	d1	d2	d3-13 during admission*	d14 (+/-2d) or discharge	wk4 (+/-5d
In- /Exclusion Criteria	x						
Patient Information and Informed Consent	x						
Randomisation	x						
Demographics		x					
Medical History and Physical Examination		x				x	
Vital Signs including Respiratory Rate[9]		x	x	x	x	x	
Laboratory Tests (blood)		x	x		x[3]	x	
Virology testing		x[2]			x[2]	x[2]	
Administer Study Medication[4]		x	x	x	x		
Assessment of WHO ordinal scale		x	x	x	x	x	x
Assessment of ICU admission and/or mechanical ventilation		x	x	x	x	x	x
Laboratory assessment in detail:							
Routine full blood count		x[5]	x		x[3]	x	
Routine coagulation studies		x[5]	x		x[3]	x	
Routine biochemistry including LDH		x[5]	x		x[3]	x	
Serum pregnancy test	x[6]						
Ferritin, IL-6, D-Dimer		x[5]	x[11]		x[3]	x	
C1INH concentration[7]		x	x			x	
Additional samples in Basel[10]:		x					
Complement proteins (e.g. C4d, C5a)		x	x		x[3]	x	
Inflammatory cytokines (e.g. IL-10)		x	x		x[3]	x	
C1INH concentration and activity		x[8]	x[8]		x (d3, 7, 10)	x	
Contact activation and coagulation proteins (e.g. factor XII)		x	x		x		
Urinary markers of renal injury		x	x		x[3]	x	
Blood volume (ml), total/study specific (Basel[10])	5/5 (5)	12/12 (22)	12/12 (22)		12/5 (10)	12/12 (17)	
Urine volume (ml), study specific in Basel[10]		5	5		5	5	
(Serious) Adverse Events		x	x	x	x	x	x

[1]Eligibility criteria have to be assessed within 72 hours after hospital admission. Randomisation may occur on the day of hospital admission.

[2]Monitoring of SARS-CoV-2 viral load will be performed in Basel and optional in other centers. This will include performance of a quantitative SARS-CoV-2 PCR from upper or lower respiratory tract at baseline and subsequently once between day 4-7 (according to local standard) and again on day 14 if participants are still admitted or between day 10-13 if discharged earlier. Standard operating procedures include the performance of a second swab following a first negative swab.

[3]Routine laboratory tests will be performed on days 3, 5, 7, and 10 during hospital admission according to standard procedures of the study site, but will include a set of hematology, coagulation and biochemistry parameters. Additional blood samples will be collected at the same time.

[4]IMP will be administered during a 64 hour period (+/-4 hours). Trial medication will be stopped in participants that are discharged or transferred to another facility.

[5]Can be omitted if results are available from the same day.

[6]A pregnancy test will be performed in women who may become pregnant.

[7]Collection of samples will be before 1st administration of study medication and subsequently at least 4 hours after the administration of study medication.

[8]Collection of samples will be before 1st administration, 10 (+/-5) minutes after the 1st administration (day 0) and before and 10 (+/-5) minutes after one administration on day 1.

[9]The worst value recorded on a given day will be documented.

[10]optional for other centers depending on their ability to collect, centrifuge, aliquot and store blood and urine samples at -80°C.

[11]IL-6 will not be measured on day 1.

* If the patient was already discharged before day 7, the assessment of the WHO ordinal scale will be conducted via a short telephone interview (Score 1 or 2).

1. STUDY ADMINISTRATIVE STRUCTURE

1.1 Sponsor, Sponsor-Investigator

Sponsor-Investigator: PD Dr. med. Michael Osthoff, University Hospital Basel, Division of Internal Medicine, Petersgraben 4, 4031 Basel, Tel. +41 61 328 6828. Fax +41 61 265 4722. Email michael.osthoff@usb.ch.

Dr. Michael Osthoff designed the study and will be responsible for data collection, study management, data analysis and interpretation and writing of the report.

1.2 Principal Investigator(s)

Basel, Switzerland: PD Dr. med. Michael Osthoff, University Hospital Basel, Division of Internal Medicine, Petersgraben 4, 4031 Basel, Tel. +41 61 328 6828. Fax +41 61 265 4722. Email michael.osthoff@usb.ch.

Local representatives at further study sites:

Zurich, Switzerland: Prof. Dr. med. Lars C. Huber, Stadtspital Triemli, Departement Innere Medizin, Birmensdorferstrasse 497; 8063 Zürich, Tel.: +41 44 416 30 02 Email: Lars.Huber@zuerich.ch

St. Gallen, Switzerland: PD Dr. med. Werner Albrich, Kantonsspital St. Gallen, Klinik für Infektiologie/Spitalhygiene , Rorschacher Strasse 95, 9007 St. Gallen, Tel.: +41 71 494 2653 Email: werner.albrich@kssg.ch

Monterrey, Mexico: Dr. Med. Adrian Camacho Ortiz, Hospital Universitario „Dr. José Eleiterio González", Infectology department, Av, Francisco I. Madero y Gonzalitos S/N, Colinia Mitras Centro, C.P 64460, Monterey, Nuevo Leon Mexico, Tel.+52 8112635696, Email: acamacho_md@yahoo.com

São Paulo, Brazil: Dr. Marcelo Rodriguez Bacci, Práxis Pesquisa Medica, alameda Francisco Alves n 169 room 193 Bairro Jardim , Santo André - São Paulo Brazil . ZIP 09090-790, Tel. +55 11 981937005 Email : mrbacci@yahoo.com

1.3 Statistician ("Biostatistician")

Dipl.-Stat. Michael Bulitta, CRM Biometrics GmbH, Marie-Curie-Str. 2, 53359 Rheinbach, Germany, Tel. +49 22 269 098 425, email: m.bulitta@crmb.de

1.4 Laboratory

1. Department of Laboratory Medicine, Head Prof. Dr. sc. nat. Katharina Rentsch, University Hospital Basel, Petersgraben 4, 4031 Basel. Tel. +41 61 265 4236, email katharina.rentsch@usb.ch.

2. Clinical Immunology Laboratory, Head Prof. Dr. med. Marten Trendelenburg, Department of Biomedicine, University Hospital Basel, Petersgraben 4, 4031 Basel. Tel. +41 61 328 6832, email marten.trendelenburg@usb.ch. Prof. Dr. med. Marten Trendelenburg is a Sub-Investigator of the trial, and the PI is a member of the Clinical Immunology Laboratory.

3. Local laboratories at the trial sites.

1.5 Monitoring institution

Clinical Trial Unit, University Hospital Basel, Schanzenstrasse 55, 4031 Basel, Switzerland.

1.6 Data Safety Monitoring Committee

We do not expect serious adverse events related to conestat alfa not mentioned in this document or not previously described in clinical studies of conestat alfa (see current Investigator's Brochure). As the frequency of comorbidities in the patient population under study is significant and clinical complications

are common in patients with COVID-19 infection, serious adverse events are possible in this setting. Hence, we will establish periodic interim safety review meetings (ISRM) by an independent committee. This Data Safety Monitoring Board (DSMB) will be responsible for safeguarding the interests of the study participants by monitoring adverse events and in particular serious adverse events. The DSMB may also consider factors external to the study when relevant information becomes available, such as scientific or therapeutic developments that may have an impact on the safety of the patients or the ethics of the study. An independent statistician from the CTU will provide the DSMB with the pertinent safety data. The DSMB's responsibility, roles and procedures will be specified in a DSMB-Charter. A first ISRM will take place after inclusion of 30 patients. The advice(s) of the (DSMB) will only be sent to the Sponsor of the study. Should the Sponsor decide not to fully implement this advice, the Sponsor will send the advice to the competent authorities and (C)EC, as appropriate, including a note to substantiate why (part of) the advice of the DSMB will not be followed.

1.7 Any other relevant Committee, Person, Organisation, Institution

Data management: Clinical Trial Unit, University Hospital Basel, Schanzenstrasse 55, 4031 Basel

IMP storage/dispensing: Pharmacy, University Hospital Basel, Spitalstrasse 26, 4031 Basel.

Pharming Technologies B.V.: manufacturer of conestat alfa, Darwinweg 24, 2333CR Leiden, The Netherlands.

2. ETHICAL AND REGULATORY ASPECTS

Infection with SARS-CoV-2 is associated with moderate to severe disease requiring hospital admission in at least 20% of patients, and consequently about 20% of these admitted patients will deteriorate despite supportive treatment. Currently, evidence regarding specific therapies to treat SARS-CoV-2 or its associated inflammatory damage is still lacking. The current study will evaluate the safety and efficacy of conestat alfa treatment in COVID-19 patients at risk for clinical deterioration.

Although experimental data indicate a potential effect of conestat alfa, it remains unclear if recruited subjects will receive any immediate benefit from this research. However, the knowledge gained from this study may help develop effective treatment strategies for patients at high risk for clinical deterioration, which is important given the paucity of available effective treatment options.

All participants will receive standard supportive care established at the sites which may include off-label use of certain drugs that are deemed to be effective in COVID-19 (e.g. hydroxychloroquine, lopinavir/ritonavir). In addition, escalation of treatment with off-label use of certain drugs (e.g. tocilizumab) in deteriorating participants is allowed during the trial. In terms of risk, conestat alfa, a recombinant human protein, has a very favourable side effect profile and major adverse events attributable to study medication are not expected in this study. Anaphylactic reactions are exceptionally rare and have only been observed in a single patient with a pre-existent allergy to rabbits. Although patients with rabbit allergy will be excluded from participating in the study, included subjects will be monitored for anaphylactic events during and at least for at least 12 hours after the last administration of conestat alfa. A randomized, open-label study design was chosen due to the unknown effect of conestat alfa in this setting. The majority of study assessments will be conducted whilst the participants are admitted with just one telephone interview, and the amount of extra blood taken is modest.

Participants' confidentiality will be maintained throughout the trial.

The decision of the CEC and Swissmedic/foreign competent authority concerning the conduct of the study will be made in writing to the Sponsor-Investigator before commencement of this study. The clinical study can only begin once approval from all required authorities has been received. Any additional requirements imposed by the authorities shall be implemented.

2.1 Study registration

The study has been registered at ClinicalTrial.gov (NCT04414631) and in the Swiss Federal Complementary Database (SNCTP) (SNCTP000003972) and in the EUDRACT Number 2020-002520-36.

2.2 Categorisation of study

Clinical trial of medicinal product, category C investigating an IMP that is not authorised in Switzerland. Conestat alfa is authorized in the European Union and the United States for the substitution treatment of hereditary angioedema.

2.3 Competent Ethics Committee (CEC)

The principal investigator and the responsible investigator at each site ensures that approval from an appropriately constituted CEC will be sought for the clinical study prior to commencement of the study. No changes will be made to the protocol without prior CEC approval, except where necessary to eliminate apparent immediate hazards to study participants. Reporting duties: Written progress reports will be sent to the CEC annually, or more frequently if requested by the CEC. All Suspected Unexpected Serious Adverse Reactions will be reported within 7 (leading to death) or 15 (others) days to the CEC. All SAEs leading to death will be reported within 7 days to the CEC. Unanticipated problems involving risks to humans will be reported immediately to the CEC.

Premature study end or interruption of the study is reported within 15 days. The regular end of the study is reported to the CEC within 90 days, the final study report shall be submitted within one year after study end. Amendments are reported according to chapter 2.10.

2.4 Competent Authorities (CA)

The Sponsor-Investigator will obtain approval from all Competent Authorities (CA) before the start of the clinical trial. Written progress reports will be sent to the CA of the concerned Member States annually,

or more frequently if requested. Unanticipated problems involving risks to humans will be reported immediately to Swissmedic and other CA of the concerned Member States.

Any changes in the research activity and all unanticipated problems involving risk to humans will be reported to the CA. Premature study end or interruption of the study is reported within 15 days. The regular end of the study is reported to the CEC within 90 days, the final study report shall be submitted within one year after study end. Amendments are reported according to chapter 2.10., non-substantial amendments will be reported as soon as possible. Additional requirements by foreign CAs will be observed.

2.5 Ethical Conduct of the Study

The study will be carried out in accordance to the protocol and with principles enunciated in the current version of the Declaration of Helsinki, the guidelines of Good Clinical Practice (GCP) issued by ICH, the Swiss Law and Swiss regulatory authority's requirements, the EU Clinical Trials Directive (EC) No.2001/20EC and in accordance with other relevant local guidelines and regulatory authority's requirements. The CEC and regulatory authorities will receive annual safety and interim reports and be informed about study stop/end in agreement with local requirements.

2.6 Declaration of interest

The study will be supported by an investigator-initiated research grant including free IMP from Pharming Technologies B.V., the manufacturer of conestat alfa. This grant is not conditioned on any pre-existing or future clinical research or business relationship between the Principal Investigator and Pharming Technologies or the University Hospital Basel and Pharming Technologies. The grant is also not conditioned on any clinical research, business relationship, or other decisions the Principal Investigator or the University Hospital Basel has made, or may make, relating to Pharming Technologies or Pharming Technologies Product. The grant will be only used to support the study.

The Principal Investigator reports having received consulting fees and a previous investigator-initiated research grant from Pharming Biotechnologies B.V.

No other conflicts of interest are reported.

2.7 Patient Information and Informed Consent

The investigators will explain to each participant the nature of the study, its purpose, the procedures involved, the expected duration, the potential risks and benefits and any discomfort it may entail. Each participant will be informed that the participation in the study is voluntary and that he/she may withdraw from the study at any time and that withdrawal of consent will not affect his/her subsequent medical assistance and treatment.

The participant must be informed that his/her medical records may be examined by authorised individuals other than their treating physician.

All participants for the study will be provided a participant information sheet and a consent form describing the study and providing sufficient information for participant to make an informed decision about their participation in the study. Participants will have enough time (at least two hours) and the opportunity to ask questions in order to decide on their participation. In the event that IC is obtained on the date that any study procedures are performed, the study record or subject´s clinical record will clearly show that IC was obtained prior to these procedures.

Due to the special situation in SARS-CoV-2 infected patients including physical isolation, consent will obtained the following way: The trial participant and the investigator will sign and date the informed consent form, that will be transferred safely out of the isolation room and stored safely until archiving is possible (usually after at least 24 hours[1]).

The formal consent of a participant, using the approved consent form, must be obtained before the participant is submitted to any study procedure.

The participant should read and consider the statement before signing and dating the informed consent form, and should be given a copy of the signed document. The consent form must also be signed and dated by the investigator (or his designee) at the same time as the participant sign, and it will be retained as part of the study records.

2.8 Participant privacy and confidentiality

The investigator and the institution affirms and upholds the principle of the participant's right to privacy and that they shall comply with applicable privacy laws. Especially, anonymity of the participants shall be guaranteed when presenting the data at scientific meetings or publishing them in scientific journals.

Individual subject medical information obtained as a result of this study is considered confidential and disclosure to third parties is prohibited. Subject confidentiality will be further ensured by utilising subject identification code numbers to correspond to treatment data in the computer files.

For data verification purposes, authorised representatives of the Sponsor (-Investigator), a competent authority (e.g. Swissmedic), or an ethics committee may require direct access to parts of the medical records relevant to the study, including participants' medical history.

2.9 Early termination of the study

The Sponsor-Investigator may terminate the study prematurely according to certain circumstances, for example:

- ethical concerns,
- insufficient participant recruitment,
- when the safety of the participants is doubtful or at risk, respectively,
- alterations in accepted clinical practice that make the continuation of a clinical trial unwise,
- early evidence of benefit or harm of the experimental intervention

A DSMB will continuously monitor patients' safety. The DSMB will independently judge and recommend on the need to halt enrolment for further evaluations or to terminate the trial based on information regarding safety.

2.10 Protocol amendments

The PI is allowed to amend the protocol. The herein mentioned local PIs and Sub-Investigators are allowed to provide suggestions for a protocol amendment as is Pharming Technologies B.V., the manufacturer of conestat alfa.

Substantial amendments are only implemented after approval of the CEC and CA respectively. Investigators will be notified immediately after approval and provided with updated study documents, and dossiers in trial registries updated accordingly.

Under emergency circumstances, deviations from the protocol to protect the rights, safety and well-being of human subjects may proceed without prior approval of the sponsor and the CEC/CA. Such deviations shall be documented and reported to the sponsor and the CEC/CA as soon as possible.

All non-substantial amendments are communicated to the CA as soon as possible if applicable and to the CEC within the Annual Safety Report (ASR).

3. BACKGROUND AND RATIONALE

3.1 Background and Rationale

On 31 December 2019, a cluster of pneumonia cases of unknown aetiology was reported in Wuhan, Hubei Province China. On 9 January 2020, China Center for Disease Control and Prevention (CDC) reported a novel coronavirus (2019-nCoV) as the causative agent of this outbreak, which is phylogenetically in the Severe Acute Respiratory Syndrome (SARS) coronarviruses (CoV) clade. It rapidly spread, resulting in an epidemic throughout China, followed by an increasing number of cases in other countries throughout the world. In February 2020, the World Health Organization (WHO) designated the disease COVID-19, which stands for coronavirus disease 2019. The virus that causes COVID-19 is designated severe acute respiratory syndrome coronavirus 2 (SARS-CoV-2). As of 14 April 2020 SARS-CoV-2 has spread globally with over 1’920’000 identified cases worldwide with over 119’00 deaths[2]. The WHO declared COVID-19 a global pandemic on March 2020.

SARS-CoV-2 is a single-stranded enveloped RNA virus, which targets cells through the viral structural spike protein that binds to the angiotensin-converting enzyme 2 (ACE2) receptor.

The clinical spectrum of COVID-19 ranges from asymptomatic carriers to respiratory failure requiring respiratory support in the intensive care unit (ICU). In the largest report of COVID-19 from the Chinese CDC 81% of 72’313 cases were of mild nature (stage I)[3]. However, a subgroup of 5% presented with respiratory failure and multi-organ dysfunction leading to death in half of the cases (stage III).

Figure 1 COVID-19 disease stages and potential therapeutic regimen[4]

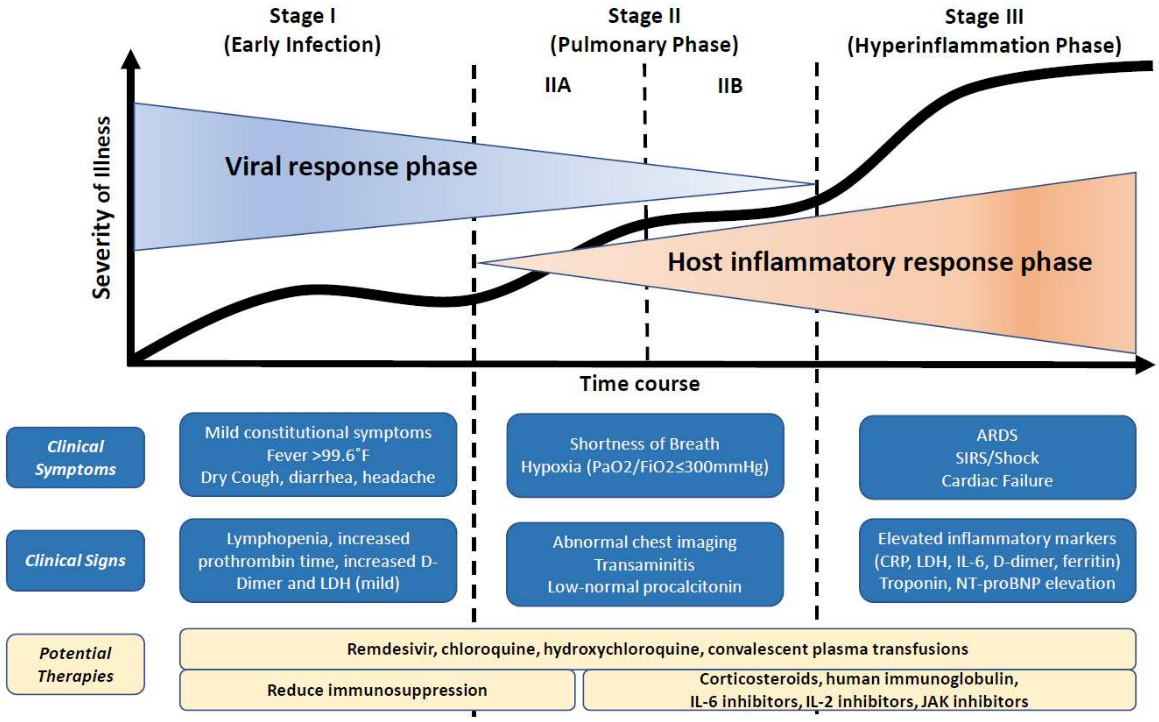

The exact reasons and factors promoting progression from stage I to stage III are not yet known. Stage I (mild infection) is characterized by infection and replication of SARS-CoV-2 in the respiratory system. In stage II (pulmonary involvement with (IIa) or without (IIb) hypoxia) patients develop viral pneumonia which is characterized not only by local viral replication but also by a localized inflammatory response. Clinically, patients present with fever, dry cough and dyspnea. Computerized tomography (CT) scans of the chest reveal uni- or bilateral ground-glass opacities, which are related to an increased fluid content in the alveoli of the lung. Affected individuals will usually require hospitalization during this stage. About 20% of stage II patients will progress into the most severe stage of illness (stage III), which is characterized by a systemic hyperinflammatory syndrome leading to acute respiratory distress syndrome (ARDS). These patients usually require admission to the ICU for escalation of medial management including mechanical ventilation[5]. In this stage, COVID-19 infection is associated with a

decrease in suppressor and regulatory T cell counts and an extensive release of proinflammatory cytokines and biomarkers such as interleukin (IL)-2, IL-6, ferritin, and D-dimer[6, 7], called a “cytokine storm”, which is thought to be the major driver of severe pneumonia caused by SARS-CoV-2.

No proven or approved effective treatment for COVID-19 infection currently exist. Several treatment candidates targeting the virus itself or systemic inflammation are under investigation in ongoing randomized trials. Given the high mortality rate in a subgroup of patients, there is clearly a need to develop management strategies to interrupt progression from stage I or II to stage III in COVID-19. The mechanism responsible for virus-induced hyper-activation of the host immune system remains poorly understood but likely involves several immune cells and inflammatory plasmatic cascades such as the complement and the kinin-kallikrein (KK) system.

The complement system (CS) is an integral part of the innate immune system and consists of a number of distinct plasma proteins that act as a first line of defence inducing an inflammatory response after opsonisation of pathogens and dying cells[8, 9]. Inflammatory responses include the activation of macrophages, neutrophils, platelets and endothelial cells, interacting with other plasmatic cascades such as the coagulation cascade and direct cell injury, thereby increasing vascular permeability and tissue injury. The CS and particularly the lectin pathway of complement has been found to interact with and be involved in the clearance of several viruses [10-14]. While the CS does not seem to be critical for controlling CoV replication, unregulated complement activation - induced by viruses including influenza and CoV - plays a crucial role in the pathogenesis of acute lung injury (ALI). Indeed, an animal model suggests that the CS mediates SARS-CoV-induced lung disease and regulates the proinflammatory response. Complement deficient mice infected with SARS-CoV were affected less severely and showed a reduced lung involvement and lower local and systemic cytokine levels compared to control mice[15]. In line, inhibition of complement C5a signalling alleviated lung damage in animal infection models using MERS-CoV [16] and an influenza H7N9[17] . Similar results were reported with inhibition of complement cascade C3a in an animal infection model using avian influenza. [18]. Recently, Gao T et al. investigated the interaction of MERS-CoV, SARS-CoV and SARS-CoV-2 with the lectin pathway of complement in more detail[19]. They demonstrate an interaction of these highly pathogenic CoV with mannose-binding lectin associated serine protease-2 (MASP-2), the key activator of the lectin pathway of complement[20], leading to uncontrolled activation of the complement cascade. In line, MASP-2 knock-out mice and mice treated with MASP-2 inhibitors showed significantly milder symptoms in a virus protein mouse pneumonia model. Mannose-binding lectin, the pattern recognition molecule of the lectin pathway, that activates MASP-2 upon binding to pathogens, was found to bind to SARS-CoV spike glycoprotein[21]. Lastly, autopsy findings from a subgroup of patients with severe COVID-19 infection revealed excessive complement activation in the lung tissue associated with complement mediated microthrombotic disease[22]. Again, the lectin pathway of complement was implicated as major complement pathway in these patients. In summary, an over-activated complement systems, driven by the lectin pathway in particular, seems to contribute to ALI in response to infection with CoV such as SARS-CoV-2 leading to the clinical picture of stage III COVID-19 infection and consequently to ARDS.

Besides the CS, the KK system may be involved in the pathogenesis of COVID-19, too. The KK system is a plasmatic cascade that after activation (shear stress of vessels, e.g. during vascular inflammation) and subsequent cleavage of kininogen by kallikrein releases bradykinin. Bradykinin binds to B2-receptors on endothelial cells leading to capillary leakage and angioedema. After enzymatic degradation bradykinin products may also bind to B1-receptors on endothelial cells that are upregulated under proinflammatory conditions and have strong vasopermeable capacity. Although direct evidence is lacking, several facts argue for an involvement of bradykinin in pulmonary angioedema observed in COVID-19. ACE2 is not only a cell membrane bound protein that is utilized by SARS-Co-V-2 to enter the cells but also possesses enzymatic activity inactivating bradykinin degradation products thereby preventing the activation of B1 receptor on endothelial cells. Interestingly, expression of ACE2 and its enzymatic activity is decreased in SARS-CoV and inflammatory conditions[23-25] and hence one may speculate that the interaction of SARS-CoV-2 with ACE2 may impair the function of ACE2 leading to a relative abundance of bradykinin degradation productions with subsequent B1 activation and local pulmonary edema[23]. Interestingly, there is a strong interaction between the CS and the KK system. For example, MASP-1, the potentiator of lectin pathway activation by MASP-2, was found to upregulate B2 receptors on endothelial cells[26]. Moreover, bradykinin release by MASP-1 mediated cleavage of kininogen was demonstrated[27]. Lastly, the enzyme that degrades bradykinin is identical to the inactivator of the complement anaphylatoxins C3a and C5a[28]. In summary, the KK system may be involved in COVID-19, particularly in stage II and III contributing to pulmonary edema and subsequently ARDS.

Apart from lung injury, COVID-19 related damage was observed in other organs such as the kidneys[29].

Of note, COVID-19-associated renal injury is not primarily mediated by viral infection, but rather by the inflammatory host response resulting in endothelial cell inflammation and microthrombosis[30]. Again, the complement system has been implicated in pre-print report (Diao B et al., preprint posted online April 10, 2020. medRxiv doi:10.1101/2020.03.04.20031120) showing strong deposition of complement components on renal tubules in an autopsy series. Collectively, these results indicate that the complement system is strongly activated in the lungs but also in the kidneys of COVID-19 patients.

C1 esterase inhibitor (C1INH) is a member of the serpin superfamily of serine-protease inhibitors. It is an acute-phase protein that has manifold targets and biological functions, including inhibition of leucocytes and interactions with endothelial cells and microorganisms. C1INH is a strong inhibitor of the CS, factor XII and plasma kallikrein of the KK system. In particular, C1INH is the natural inhibitor of the lectin pathway of complement. MASP-1 and-2 seem to be the major target of C1INH with less effective inhibition of the classical pathway of the CS[31]. Decreased plasmatic antigenic levels of C1INH result in uncontrolled production of vasoactive peptides, which leads to the characteristic episodes of local soft tissue swelling observed in hereditary angioedema (HAE)[32]. C1INH deficiency seems to cause uncontrolled activation of MASP-1, which may aggravate HAE [33]. Currently, three C1INH preparations are available, two of them plasma-derived and one recombinant, i.e., rhC1INH (conestat alfa, Ruconest®, Pharming, Leiden, The Netherlands). Conestat alfa shares an identical protein structure with plasma-derived C1INH (pdC1INH) but has a different glycosylation pattern (containing abundant oligomannose residues), which is responsible for a shorter half-life than pdC1INH (3h vs. 30h) [34, 35]. Although comparable inhibition for most target proteases was demonstrated (including C1s, Factor XIa, XIIa and kallikrein) [35], conestat alfa seems to target the activation of the lectin pathway more effectively compared to plasma-derived preparations [36]. Despite the broad interference with several cascades and targets, major adverse events or unique toxicities have not been demonstrated in previous studies with the exception of a potential risk of allergic reactions in patients with rabbit dander allergy.

Although data on C1INH treatment in the context of SARS or influenza infections are lacking, results from pilot studies suggest that C1INH treatment may reduce the collateral damage caused by hyperinflammation in human sepsis[37-39]. Also, reduced occurrence of capillary leakage after allogeneic stem cell transplantation has been observed in a further study[40]. C1INH was also able to block MASP-2 mediated overactivation of the complement system induced by several CoVs[19]. With regards to renal injury previous experimental and human data point to a protective effect of conestat alfa in the setting of renal injury caused by inflammation[41, 42].

In the context of COVID-19, conestat alfa treatment may 1) dampen uncontrolled complement activation and collateral lung and renal damage by inhibiting MASP-1 and MASP-2 in addition to classical pathway activation and 2) reduce capillary leakage and subsequent pulmonary edema by direct inhibition of kallikrein activity and subsequent bradykinin release, inhibition of MASP-1 mediated upregulation of B2 receptors on endothelial cells and potentially reduced upregulation of B1 receptors on endothelial cells as a consequence of a reduced complement mediated inflammatory response.

Rationale of the study

The rationale for conestat alfa is based on its mode of action, in particular its anti-inflammatory properties by inhibiting the CS and the KK system. Given the high percentage of sequence homology between SARS-CoV and SARS-CoV-2 similar complement and KK system dependent mechanisms may be involved in the susceptibility and severity of COVID-19[43]. Both plasmatic cascades most likely contribute to the inflammatory response after SARS-CoV-2 infection leading to ALI, ARDS and potentially death.

Given the above mentioned evidence, the lack of approved and effective treatment options for COVID-19, in particular for patients at risk of progression from stage II to stage III, the involvement of the CS and the KK system in ALI after infection with CoV and other respiratory viruses and the potent inhibition of both cascaded by conestat alfa, a human trial to explore the effectiveness of conestat alfa in preventing the progression of COVID-19 infection in hospitalized non-critically ill patients is clearly desirable. The primary purpose of this study is to evaluate if adding conestat alfa to standard of care (SOC) in patients admitted for stage II COVID-19 infection may reduce the risk of disease progression, i.e. ALI requiring mechanical ventilation, or increase the chance of a faster clinical improvement compared to SOC alone. The treatment regimen is designed based on the timing and cause of the lung injury in COVID-19 infection and the role of the CS and KK system in the pathophysiology of COVID-19.

3.2 Investigational Product (treatment, device) and Indication

Conestat alfa is purified from the milk of rabbits expressing the gene coding for human C1-INH.

Conestat alfa is supplied as a sterile, preservative-free, white/off-white lyophilized powder for reconstitution for injection. Each vial contains 2100 units of conestat alfa. After reconstitution with 14 mL of sterile water for injection, each vial contains 150 U of conestat alfa per 1 mL in a 20 mM sodium citrate buffer with a pH of 6.8; vials are for single use only. The drug's shelf-life is 48 months at ≤25°C.

For further information please refer to the Investigator's Brochure (IB).

Conestat alfa is licensed by the European Medicines Agency (EMA) and the United States Food and Drug Association (FDA) for the treatment of acute angioedema attacks in adults with hereditary angioedema (HAE) due to C1-INH deficiency. It is not yet licensed for any indication in Switzerland.

3.3 Preclinical Evidence

The inhibitory potency of conestat alfa towards the target proteases C1s, kallikrein, factor XIa and factor XIIa was found to be comparable with the inhibitory potency of plasma-derived C1-INH, tested *in vitro*. In addition, although C1-INH can inhibit plasmin, tPA and thrombin, inhibition is week. The expected increases of C1-INH plasma concentrations in the proposed clinical study do not have a significant effect on the activity of the proteases and are unlikely to affect the balance between clotting and fibrinolysis. Please refer to the IB for further information.

In the nonclinical program no concerns were found with respect to tolerability, cardio-respiratory safety, embryofetal toxicity, local tolerance, or immunogenicity of conestat alfa.

Acute and repeat dose toxicology and safety pharmacology as well as local tolerance studies in rats, dogs and rabbits support the safety-in-use of the conestat alfa preparation.

The acute studies showed no overt toxicological effects in rats and dogs with a single dose up to 1250 U/kg. A No-Observed-Adverse-Effect-Level (NOAEL) was established at 625 U/kg when dosed by continuous IV infusions for 14 days in rats and by daily IV infusions (5 mL/min) for 5 days in dogs. In the 14-day pivotal repeat dose toxicity study in monkeys, the NOAEL was established at twice daily IV infusions of 1000 U/kg.

The total dose of 2000 U/kg/day in this monkey study given for 14 consecutive days, exceeds the intended total clinical doses of 150-300 U/kg given a day by 7 to 13-fold without adverse effects.

In a safety pharmacology study in dogs (dosed at 625 U/kg, i.e. 12.5- to 4-fold the intended total clinical dose of 50-150 U/kg), no overt effects on vital functions such as the cardiovascular or respiratory system were observed. The established NOAEL was 625 U/kg.

Conestat alfa is predominantly cleared from the circulation by liver receptors. It is more rapidly cleared from the circulation in rats, dogs and cynomolgus monkeys in comparison with plasma-derived C1-INH. This faster clearance likely results from the differential glycosylation, including a lower degree of sialylation in conestat alfa. Degradation is expected to be by proteolysis/hydrolysis within intracellular lysosomes. From studies in rats, dogs, and cynomolgus monkeys, it can be concluded that conestat alfa distribution is limited to the vascular compartment. For further information, see the IB.

Conestat alfa has not been assessed in preclinical models of COVID-19 or related CoV infections. However, pdC1INH treatment was shown to reduce the activation of the CS and KK system in a large-animal model of septic shock. Importantly, release of proinflammatory cytokines such as TNF-α and IL-6 was attenuated [44]. In addition, administration of conestat alfa reduced lung tissue damage in an animal model of hemorrhage induced systemic inflammation. In particular, conestat alfa decreased tissue complement activation and deposition and circulating proinflammatory cytokines[45].

3.4 Clinical Evidence to Date

Conestat alfa has not been assessed in patients with COVID-19 or related CoV infections.

Conestat alfa has been extensively investigated in the treatment of acute attacks in patients with hereditary angioedema having demonstrated efficacy and safety (please, see IB for further details). In addition, a recent randomized, double-blind, placebo-controlled pilot study has demonstrated its efficacy regarding attenuation of acute renal injury in patients undergoing elective coronary angiography[42].

With regards to infection and inflammation, pdC1INH treatment reduced the collateral damage caused by hyperinflammation in human sepsis in several small pilot studies[37-39]. In particular, it decreased complement and neutrophil activation[46]. Also, a reduced occurrence of capillary leakage after allogeneic

stem cell transplantation has been observed in a further study in line with a decrease in the complement C5 activation product C5a[40, 47].

We have recently treated five patients admitted with stage II COVID-19 infection with compassionate use conestat alfa in addition to SOC in order to limit pulmonary inflammation and subsequent deterioration. Conestat alfa was administered 12-hourly over 48 hours starting with a loading dose of 8400 U and continuing with 4200 U. Conestat alfa was well tolerated with no treatment-emergent adverse events. Three patients had an immediate favourable response with rapid defervescence and decline in inflammatory markers and were discharged within a week. Two patients were administered additional treatment with tocilizumab and recovered afterwards, although one of these two patient rapidly progressed after 24 hours of treatment requiring transient mechanical ventilation.

3.5 Dose Rationale / Medical Device: Rationale for the intended purpose in study (pre-market MD)

Pharmacological characteristics

A phase 1, dose-escalating study in 12 asymptomatic patients with HAE demonstrated that conestat alfa administration provides a dose-dependent restoration of complement homeostasis. Patients with functional C1-INH levels <40% and C4 levels <300 µg/mL (values expectable in patients with HAE) received 2 IV doses of conestat alfa (range 6.25 to 100 U/kg), with a washout period of ≥5 weeks.

A dose-dependent, biologic activity was demonstrated by increased plasmatic levels of C4, which is a substrate for activated C1s: 12 hours after conestat alfa administration (100 U/kg), the mean plasma levels of C4 were approximately double compared to baseline.

Comparable results were shown after the 50 U/kg dose of conestat alfa, which was associated with a maximum C4 level of approximately 1.76-fold that at baseline, achieved after 10.7 hours.

A pharmacokinetic modelling indicated that a single IV injection of conestat alfa at a dose of 50 U/kg restores functional C1-INH levels to at least the lower limit of the normal range in almost all patients[48].

Following the slow IV administration (15 min) of 50 U/kg of conestat alfa to 6 asymptomatic patients with HAE, the mean maximum plasmatic level of functional C1-INH was 1.36 U/mL, mean volume of distribution was similar to plasma volume (approximately 3 L), mean elimination half-life was 2.4 hours and clearance was approximately 23 mL/min[49].

The pharmacokinetic data derived from the phase 1 studies demonstrated that the half-life of conestat alfa is shorter than plasma-derived C1-INH which is due to the different glycosylation of conestat alfa, leading to more rapid hepatic clearance compared to plasma-derived C1-INH.

In a phase 1 study in healthy volunteers, patients were administered with conestat alfa on 5 occasions with intervals of at least 3 weeks. 14 patients received a total of 59 administrations of conestat alfa at a dosage of 100 U/kg. The mean elimination half-life was 2.7 hours and the baseline corrected Cmax was 2.6 U/mL. These values were comparable to those estimated in other studies.

The course of functional C1-INH levels after infusion of 100 U/kg of conestat alfa was identical after 1st, 3rd and 5th administration.

Conestat alfa is cleared via mannose/asialoglycoprotein receptors on macrophages and hepatic cells with carbohydrate recognition. Conestat alfa is not expected to be excreted, but to be fully eliminated by degradation. As there are no clinical data with conestat alfa in patients with hepatic impairment, and as hepatic impairment may prolong the plasma half-life of conestat alfa, patients with liver cirrhosis will be excluded from study participation.

Safety

The safety data analyses demonstrate that conestat alfa at doses of 50 and 100 U/kg are generally safe and well tolerated when administered for treatment and prevention of acute attacks in patients with HAE[50].

The adverse event profile found in the randomized, placebo-controlled studies was similar for patients treated in the conestat alfa and saline treatment groups.

The use of the product in patients allergic or suspected to be allergic to rabbits should be avoided because of the risk of an allergic reaction.

Throughout the clinical development, no safety signal related to hematology, biochemistry, coagulation,

urinalysis, vital signs, or ECG parameters was noted.

Evidence regarding a thrombogenic risk of conestat alfa, has not been recorded during clinical development and clinical studies. Conestat alfa had no effect on activation of coagulation and fibrinolysis[51].

Because conestat alfa is derived from the milk of transgenic rabbits and contains a small percentage (0.002%) of Host-Related Impurities (HRI), immunogenicity was extensively tested throughout the clinical development programs, leading to the evidence that conestat alfa has low potential to induce anti-C1-INH antibodies or anti-HRI response.

Sporadic, transient immune responses to conestat alfa and HRI were observed, but with no associated clinical findings. No patient developed neutralizing antibodies to C1-INH. No impact of immunogenicity on clinical efficacy or safety was observed.

There was no plausible temporal association between treatment-emergent adverse events or new acute attacks and the presence of any confirmed anti-C1-INH or anti-HRI antibodies in patients with HAE.

No patients tested positive for neutralizing antibodies to endogenous C1-INH or conestat alfa and no induced anti-rabbit IgE antibodies were reported. Healthy patients with clinically manifest rabbit allergy were administered conestat alfa by skin prick and – in case of lack of allergic response – an intradermal and subcutaneous injection. Two out of twenty patients revealed a positive response, suggesting that even in patients with a pre-existing allergy to rabbits the risk of a hypersensitivity reaction upon administration of conestat alfa is limited.

Lastly, safety was favourable with no related or treatment-emergent adverse events in a recent double-blind, placebo-controlled trial in elderly patients undergoing elective coronary angiography[42].

Description and justification of route of administration and dosage

Because C1-INH is a plasma glycoprotein, an IV route of administration has been chosen for the existing indication (acute attacks in patients with HAE). The same route of administration will be used in this study.

The approved dose for treatment of acute attacks in patients with HAE is 50 U/kg (up to 4200 U), with an option to repeat the dose once based on clinical symptoms. Studies on prophylactic usage at a dose of 50 U/kg (up to 4200 U) twice weekly, up to 4 weeks, to treat patients with HAE with frequent acute attacks were also realized and appeared efficacious and well tolerated. Maximum doses (4200 U or 8400 U) have been administered to patients weighing 84 kg or more.

In the current study, conestat alfa (150 U/ml) at a dose of 8400 U and 4200 U will be administered by slow IV injection over a period of approximately 5-10 minutes in an 8-hour interval over 72 hours. The same dosage will be used for all patients irrespective of their body weight.

The dosing scheme is highlighted in Figure 2.

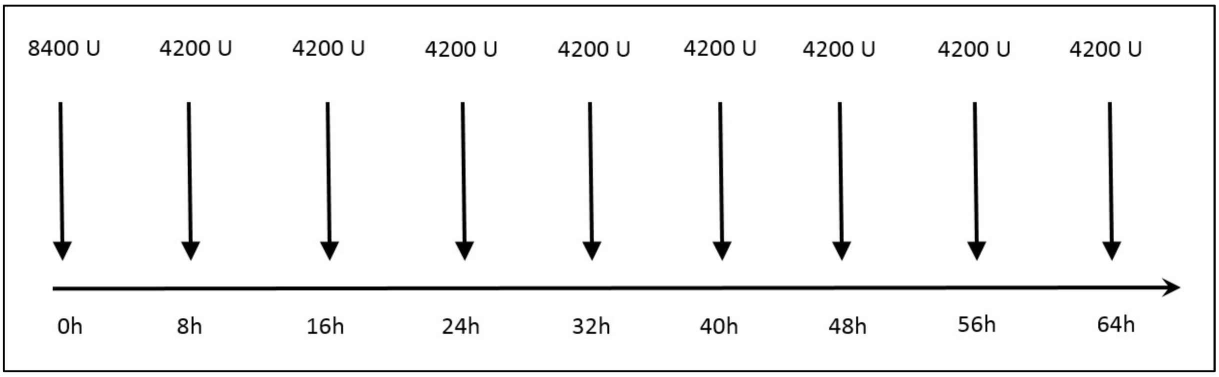

Figure 2: dosing scheme of conestat alfa during 72 hours

The dosage regimen was chosen based on the following rationale

- Although C1INH antigenic concentrations are often elevated in patients with infections (C1INH is an acute phase reactant), the functional activity may be compromised as a consequence of modified/cleaved and consequently inactive C1INH[52]. Hence, to overcome a relative deficiency, a higher loading dose was chosen. This is in line with studies of C1INH in patients

with myocardial infarction showing that only the higher dose of 100 U/kg (equal to 8400 U) was associated with an almost complete inhibition of complement activation[53].

- The half-life of conestat alfa is 2.5 hours, hence in order to achieve a sustained inhibition of the CS and KK system, repeated administration has been chosen. Despite the short half-life sustained inhibition of the target proteases may be guaranteed related to the irreversibly inhibition of target proteases followed by clearance from the bloodstream. In order to balance effectivity with practicability and potential toxicity we decided to limit the total daily dose and administer conestat alfa every 8 hours.
- Repeated administration of conestat alfa over three days has been chosen, as hyperinflammation may develop several days after the admission, in particular during the time of seroconversion (development of antibodies against SARS-CoV-2)[54]. Anti-inflammatory strategies, such as the current proposed treatment regimen with conestat alfa may have a particular role at the time of induction of hyperinflammation. Hence, treatment for at least 3 days is warranted.

Participants will either receive conestat alfa with SOC or only SOC.

3.6 Explanation for choice of comparator (or placebo)

Currently, no approved or effective treatment options for COVID-19 infections exist. Participants will either receive conestat alfa plus SOC or only SOC. SOC may include treatment for underlying comorbidities and management of COVID-19 symptoms. Importantly, local treatment protocols including drugs with perceived benefits in COVID-19 infections such as hydroxychloroquine or lopinavir/ritonavir are allowed as long as they are declared as SOC. In order to facilitate patient recruitment in a time of restricted human and financial resources and because of an objective primary endpoint, a placebo group will be omitted.

3.7 Risks / Benefits

Despite strong reasons for a benefit of conestat alfa in the setting of hyperinflammation, it is unclear if participants in the current study will receive any immediate benefit from treatment with conestat alfa. However, the knowledge gained from this study may help to better understand the pathophysiology of ALI and hyperinflammation in SARS-CoV-2 and related CoV infections. In addition, this trial may serve as pilot study for future conestat alfa studies in patients with severe infections characterized by hyperinflammation and subsequent tissue damage.

Previous experimental models have not suggested a detrimental effect of complement inhibition on viral replication, and C1INH treatment has not been associated with an increased risk for secondary bacterial or viral infections in humans.

Conestat alfa is approved for the treatment of acute angioedema attacks in adult and adolescent patients with HAE in the EEA countries and the United States of America (USA) and for adults in South Korea and Israel. Up to October 2018, 268 unique patients have been exposed to conestat alfa in the conestat alfa clinical development program. About 2400 patients have been commercially prescribed conestat alfa without any change to the risk-benefit profile.

To date, the only known relevant clinical risk associated with conestat alfa is the possibility of an allergic reaction to Host-Related Impurities (rabbit). Patients with medical history of allergy to rabbits or rabbit-derived products including conestat alfa or suspicion of such an allergy, are excluded from participation.

To minimize the risk for allergic reactions to conestat alfa, treated patients will be monitored for clinical symptoms of hypersensitivity after dosing. All treatments will be administered in the hospital and all patients will stay under close observation until discharge from the hospital.

Conestat alfa was demonstrated to be a safe option for reducing the risk of contrast-induced renal damage in high-risk patients[42]. In addition, conestat alfa was safely administered to five patients suffering from stage II COVID-19 infection at our hospital.

Expected adverse events during the study

As per last IB, expected adverse reactions are described as follows:

The clinical development program supporting safety of conestat alfa is based on a total number of 268 unique patients (up to Oct 2018). Those 268 patients received a combined total of almost 1600 administrations of conestat alfa.

The treatment emergent adverse events (TEAEs) that occurred with onset within 7 days of treatment with conestat alfa that were most frequently reported (observed in ≥3% of patients treated with conestat alfa) were headache (11%), nausea, and diarrhea (3% each). There was no evidence of an increase in the percentages of patients with TEAEs or related TEAEs, or in the seriousness or severity of TEAEs, with increases in conestat alfa dose, treatment of a larger number of attacks, and/or treatment of a single attack with an additional dose.

The ADRs listed in the European SmPC are headache (common) and vertigo, paresthesia, throat irritation, diarrhea, nausea, abdominal discomfort, oral paresthesia, urticaria, and swelling (all uncommon). As described in the US prescribing information for conestat alfa, AEs of headache, sneezing, angioedema, erythema marginatum, skin burning sensation, back pain, C-reactive protein increased, fibrin D-dimer increased, vertigo, lipoma, nausea, and diarrhea occurred in ≥2% of conestat alfa -treated patients. Note that AEs are listed in the US prescribing information regardless a causal relationship with conestat alfa.

The frequency of adverse reactions listed above is defined using the following convention:

• Very common (≥1/10),

• Common (≥1/100 to <1/10),

• Uncommon (≥1/1,000 to <1/100),

• Rare (≥1/10,000 to <1/1,000),

• Very rare (<1/10,000),

Specific antidotes against conestat alfa do not exist. In the unlikely event of an allergic reaction, standard treatment regimens indicated by the clinical situation (e.g. epinephrine, antihistamines, corticosteroids, etc.) will be administered.

The level of risk may evolve over time, during the study: consequently, a Data Safety Monitoring Board (DSMB) will monitor the safety of all patients, as well as the continuing validity and scientific merit of the study. Currently, one competing trial in COVID-19 patients (WHO Solidarity trial) is conducted at one study site (Basel).

3.8 Justification of choice of study population

Approximately 20% of patients with COVID-19 infection require hospitalization for supportive treatment. The majority of these patients will suffer from viral pneumonia visible on chest CT scans. Approximately 15-20% of these patients will progress to severe ALI requiring mechanical ventilation and ICU support. Patients with confirmed COVID-19 disease and admitted to a non-ICU ward will be eligible for the study. This population was chosen, as anti-inflammatory treatments such as conestat alfa may interfere most effectively during stage II disease, i.e. during the early inflammatory response phase. In addition, interventions that prevent patients from deteriorating and requiring mechanical ventilation are highly desired and represent an area of unmet clinical need in a pandemic situation with limited ICU and ventilation support capacity. This population offers the opportunity to impact not only on an individual but also on a population level.

4. STUDY OBJECTIVES

4.1 Overall Objective

The purpose of this study is to determine whether conestat alfa administered early during hospital admission for COVID-19 provides additional benefit compared to SOC alone with regards to the clinical course of patients by limiting the local and systemic inflammatory response, and to describe its safety profile in this population.

4.2 Primary Objective

The primary objective of the study is to determine if adding 72 hours of treatment with conestat alfa to SOC treatment in adult participants admitted with non-critically ill COVID-19 infection will affect disease severity including progression to severe disease requiring mechanical ventilation within 7 days after enrolment as assessed by the WHO Ordinal Scale for Clinical Improvement.

4.3 Secondary Objectives

To evaluate the effect of conestat alfa treatment in addition to SOC treatment compared to only SOC treatment on disease progression as measured by

- Time to clinical improvement (time from randomisation to an improvement of two points on the seven-category WHO ordinal scale or live discharge from hospital, whichever came first) within 14 days after enrolment.
- Proportion of participants alive and not having required invasive or non-invasive ventilation at 14 days after enrolment.
- Proportion of subjects with an ALI (defined by PaO_2/FiO_2 ratio of $\leq$300mmHg) within 14 days after enrolment

4.4 Safety Objectives

The study aims to assess the safety of conestat alfa treatment in patients admitted with COVID-19 infection and receiving SOC treatment including overall incidence of adverse and serious adverse events and their relationship to the study treatment during a four-week follow-up.

5. STUDY OUTCOMES

5.1 Primary Outcome

The primary endpoint will be the disease severity on the 7-point Ordinal WHO scale on day 7 (for the current study, score 0 will be omitted and score 6 and 7 will be combined). This endpoint has been suggested by WHO for clinical trials in patients with COVID-19. The ordinal scale measures illness severity over time.

Patient State	Descriptor	Score
Uninfected	No clinical or virological evidence of infection	0
Ambulatory	No limitation of activities	1
	Limitation of activities	2
Hospitalized Mild disease	Hospitalized, no oxygen therapy	3
	Oxygen by mask or nasal prongs	4
Hospitalized Severe Disease	Non-invasive ventilation or high-flow oxygen	5
	Intubation and mechanical ventilation	6
	Ventilation + additional organ support – pressors, RRT, ECMO	7
Dead	Death	8

Figure 3: WHO ordinal scale for clinical improvement. For the current study, score 0 will be omitted, and score 6 and 7 will be combined.

Patient State	Description	Score
Outpatient	No limitation in activities	1
	Limitation in activities	2
Hospitalized Mild disease	No oxygen therapy	3
	Oxygen by mask or nasal prongs	4
Hospitalized Severe disease	Non-invasive ventilation or high-flow oxygen	5
	Intubation, mechanical ventilation +/- additional organ support	6
Death	Death	7

5.2 Secondary Outcomes

The secondary endpoints will evaluate the effect of conestat alfa treatment in addition to SOC treatment relative to only SOC treatment on disease progression as measured by

- Time to clinical improvement (time from randomisation to an improvement of two points on the seven-category WHO ordinal scale or live discharge from hospital, whichever came first) within 14 days after enrolment.
- Proportion of participants alive and not having required invasive or non-invasive ventilation at 14 days after enrolment.

- Proportion of subjects with an ALI (defined by PaO_2/FiO_2 ratio of $\leq$300mmHg) within 14 days after enrolment

5.3 Other Outcomes of Interest

- Changes in the ordinal WHO scale from baseline over 14 days
- Length of hospital stay until day 28 in survivors.
- Proportion of participants progressing to mechanical ventilation on day 7 and day 14
- Proportion of participants requiring ICU treatment on day 7 and 14
- Length of ICU stay until day 28
- Ventilator-free days until day 28
- All-cause mortality (time from randomisation to death within four weeks)
- Changes in biomarker levels until day 14: CRP, LDH, D-dimer, ferritin, IL-6, lymphocyte count
- Time to virological clearance of SARS-CoV-2 by PCR from upper or lower respiratory tract samples (time from enrolment to first of 2 negative assays at least 12 hours apart)
- Proportion of patients receiving additional anti-inflammatory treatment such as tocilizumab or immunoglobulins within 14 days
- Time to defervescence (temperature <38.0°C sustained for at least 48 hours)
- Time to clinical improvement (defervescence, normalization of oxygen saturation (>93%) and respiratory rate) until day 28
- Duration of supplemental oxygen until day 28
- Incidence of acute kidney injury classified on the basis of the worst serum creatinine according to the KDIGO classification, and changes of serum creatinine and estimated glomerular filtration rate (eGFR, estimated with the CKD-EPI equation) from baseline
- In a subgroup of patients (in Basel and optional in other centers) the pharmacokinetics and pharmacodynamics of conestat alfa in COVID-19 patients will be characterized by measuring the concentration of conestat alfa and the activity of C1INH,inflammatory cytokines and proteins (such as C4d, C5a, sC5-b9, IL-10) and parameters of the contact activation and coagulation cascade (such as factor XII and pre-kallikrein). In addition, changes of urinary markers of renal injury (such as NGAL, KIM-1, osteopontin and clusterin) from baseline will be analysed.

5.4 Safety Outcomes

The study will evaluate the safety of conestat alfa in the setting of COVID-19 infections if added to SOC compared to SOC treatment only by measuring the incidence of adverse events up to 4 weeks inclusion.

6. STUDY DESIGN

6.1 General study design and justification of design

This is an investigator-initiated, exploratory, randomized, parallel-group, open-label, multi-center, phase 2 clinical study in patients with confirmed COVID-19 infection and admitted to the hospital for treatment of SARS-CoV-2 infection to estimate the effect size of conestat alfa treatment in addition to standard of care compared to only standard of care on the proportion of patients surviving without requiring mechanical ventilation.

The study will recruit approximately 120 patients (80 patients in the intervention and 40 in the control arm) from several sites in Switzerland, Brazil and Mexico. Sites will be selected on the basis of estimated numbers of cases, the availability of a committed principal site investigator and research team, and the capacity to collect samples as per protocol.

Participants will be randomly assigned in a 2:1 ratio to conestat alfa treatment in addition to standard of care or only standard of care stratified by the site. Screening, informed consent and randomisation will happen as early as possible after admission to the hospital, usually within 72 hours after admission. Participants will receive intravenous conestat alfa or no additional treatment during a 72-hour period usually starting on the day of informed consent (=day 0). Both groups will continue to receive SOC treatment for COVID-19 infection. Blood samples will be collected before and during the 72-hour administration period. Follow-up will include the period until discharge and a telephone interview at four weeks (+/- one week) if participants are discharged earlier to assess potential adverse events.

Duration of participant's participation will be 4 weeks (+/- 5 days). Screening phase duration is up to 72 hours; treatment phase is a maximum of 72 hours, and follow-up period is 4 weeks after enrolment. At least three attempts will be made to contact the participant or his person of confidence at week 4 before the participant may be considered as lost to follow-up.

6.2 Methods of minimising bias

Unmeasured confounding and bias will be minimised by randomisation and objective endpoints.

6.2.1 Randomisation

Participants will be randomized to two parallel groups in a 2:1 ratio in an open-label controlled design to receive either conestat alfa (intervention) in addition to standard of care (SOC) or SOC only (control). Randomisation will be stratified by the study site before inclusion using permuted-block randomization with varying block sizes. Allocation will be concealed, and randomization implemented via the electronic data capture system software SecuTrial®.

6.2.2 Blinding procedures

Participants, treating physicians, nurses, investigators and DSMB members will not be blinded.

6.2.3 Other methods of minimising bias

Not applicable.

6.3 Unblinding Procedures (Code break)

Not applicable.

7. STUDY POPULATION

Patients admitted for the management of confirmed COVID-19 infection will be approached. If enrolment goals are not met expansion to other centers in Switzerland, Brazil and Mexico will be explored.

7.1 Eligibility criteria

Participants fulfilling all of the following inclusion criteria are eligible for the study:

- Informed Consent as documented by signature
- Age 18-85 years
- Females or Males
- admitted to the hospital because of confirmed (by a positive SARS-CoV-2 PCR result) COVID-19 infection
- evidence of pulmonary involvement on CT scan or X-ray of the chest (e.g. ground glass opacities)
- symptom onset within the previous 10 days, i.e. fever or one respiratory symptom (patients presenting later may have already progressed to an inflammatory state that is potentially not amenable to C1INH treatment). Respiratory symptoms include cough, sore throat, hemoptysis, shortness of breath, runny nose, or chest pain.
- expected to remain an inpatient over the next three calender days from time of enrolment
- at least one additional risk factor for progression to mechanical ventilation: 1) arterial hypertension, 2) ≥50 years, 3) obesity (BMI≥30.0 kg/m^2), 4) cardiovascular disease, 5) chronic pulmonary disease, 7) chronic renal disease, 6) C-reactive protein of >35mg/L, 7) oxygen saturation at rest in ambient air of ≤94%. Cardiovascular disease includes a history of coronary artery disease, cerebrovascular disease, peripheral artery disease, rheumatic heart disease, congenital heart disease and of recent (< 3 months) deep vein thrombosis or pulmonary embolism. Chronic pulmonary disease includes a history of chronic obstructive pulmonary disease, asthma, occupational lung disease, interstitial lung disease or of pulmonary hypertension. Chronic renal disease is defined as a history of an estimated glomerular filtration rate (according to the Chronic Kidney Disease Epidemiology Collaboration equation) < 60ml/min/1.73 m^2 for at least three months.

The presence of any one of the following exclusion criteria will lead to exclusion of the participant, for example:

- Age >85 years
- Contraindications to the class of drugs under study (C1 esterase inhibitor), e.g. known hypersensitivity or allergy to class of drugs or the investigational product
- Treatment with tocilizumab or another Il-6R or Il-6 inhibitor before enrolment
- History or suspicion of allergy to rabbits
- Women who are pregnant or breast feeding
- Active or planned treatment with any other complement inhibitor
- Liver cirrhosis (any Child-Pugh score)
- Incapacity or inability to provide informed consent
- Currently admitted to an ICU or expected admission within the next 24 hours
- Currently receiving invasive or non-invasive ventilation (with the exception of high-flow oxygen therapy).
- In the opinion of the treating time, death is deemed to be imminent and inevitable within the next 24 hours
- Participation in another study with investigational drug within the 30 days preceding and during the present study with the following exemptions: 1) participation in COVID-19 drug trials started at least 48 hours before admission (e.g. postexposure prophylaxis with hydroxychloroquine) and 2) participation in COVID-19 drug trials during ICU admission
- Previous enrolment into the current study
- Enrolment of the investigator, his/her family members, employees and other dependent persons

7.2 Recruitment and screening

Patients who are admitted to the hospital for treatment of confirmed COVID-19 infection will be screened after results from history (symptom onset) and routine investigations (including a CT scan or X-ray of the chest) are available. Patients that at least fulfil the age, CT/X-ray chest criteria, positive SARS-CoV-2 PCR and reason for admission will be approached by a study doctor to explain the study, verify inclusion/exclusion criteria and obtain informed consent. Participants will have at least two hours and the opportunity to ask questions in order to decide on their participation

7.3 Assignment to study groups

Following study enrolment, an investigator or study nurse will determine treatment allocation via the electronic data management system, stratified according to the study site. Subsequently, conestat alfa will be supplied by the local pharmacy to the respective ward and can then be prepared by a regular ward nurse for administration in the active treatment group. The pharmacy of the University Hospital Basel will distribute conestat alfa within Switzerland. The Sponsor-Investigator will organize the distribution of the drug to non-Swiss sites in collaboration with the drug manufacturer Pharming Technologies B.V.

7.4 Criteria for withdrawal / discontinuation of participants

Subjects may voluntarily withdraw from study participation at any time without having to provide a reason. Subjects may be withdrawn because of the appearance of a new health condition requiring care or medications prohibited by the protocol, unacceptable adverse event, refusal to continue treatment, or at the Investigator's discretion if it is in the subject's best interest.

A subject who withdraws informed consent before randomization or who develops a violation of the selection criteria before randomization is defined as a screening failure. No follow-up of screening failures will be performed.

Participants who withdraw informed consent, who do not fulfil inclusion/exclusion criteria after obtaining informed consent AND have not received any study medication will be withdrawn from the study.

Participants who experience a type I allergic reaction after any dose of study medication will be discontinued from further study interventions. Withdrawn or discontinued participants will not be replaced. Follow-up for all patient groups will be explained in Section 9.2.5.

8. STUDY INTERVENTION

8.1 Identity of Investigational Products (treatment / medical device)

8.1.1 Experimental Intervention (treatment)

Conestat alfa (Ruconest®) will be supplied by the production company Pharming Technologies, B.V., the Netherlands. Conestat alfa is a recombinant analogue of human C1INH for intravenous injection. The primary and secondary structures of the molecule and target protease selectivity are consistent with those of plasma-derived C1INH. Conestat alfa is purified from the milk of transgenic rabbits, and supplied as a sterile, preservative-free, white/off-white lyophilized powder for reconstitution for injection. Conestat alfa contains less than 0.002% of rabbit-related impurities. One international unit (U) of Conestat alfa activity is defined as the equivalent of C1INH activity present in 1 mL of pooled normal plasma. Each vial of Conestat alfa contains 2100 U of Conestat alfa, 937 mg of sucrose, 83.3 mg of sodium citrate dihydrate and 1.0 mg of citric acid monohydrate. After reconstitution with 14 mL of sterile water for injection, each vial of Conestat alfa contains 150 U of Conestat alfa per 1 mL in a 20 mM sodium citrate buffer with a pH of 6.8. Conestat does not contain preservatives and each vial is for single use only. After randomization, an study nurse will open the respective sealed boxes, will reconstitute the respective number of conestat alfa vials and prepare the study medication for the intervention group. The control group will not receive any study medication. Conestat alfa is for intravenous use only. The reconstituted solution is administered as a slow intravenous injection over approximately ten minutes. Recommended doses of Conestat alfa for the treatment of an acute angioedema attack are 50 U/kg if body weight <84kg and 4200 U if >84kg. A second dose may be administered at the same recommended dose level within a 24 hour period. The experimental intervention in our study does deviate from the Summary of Product characteristics regarding dosing of the licensed product. In addition, we will not use the product according to the licensed indication (FDA and EMA). A market batch will be used for the study.

8.1.2 Control Intervention (standard/routine/comparator treatment / medical device)

Participants in the control arm will receive SOC treatment according to local guidelines.

8.1.3 Packaging, Labelling and Supply (re-supply)

The investigational product (conestat alfa) is supplied in single-use 25ml glass vials with a stopper (siliconized chlorobutyl rubber) and a flip-off seal (aluminium and colored plastic). Each carton contains one single-use vial. Sufficient amounts of conestat alfa will be supplied by the manufacturer Pharming Technologies B.V. to the pharmacy of the University Hospital Basel prior to study commencement. Both the carton and the glass vial of conestat alfa are labelled as "Ruconest® 2100 U, powder for solution for injection, conestat alfa. For intravenous use only". One vial contains 2100 U of conestat alfa, corresponding to 2100 U/14 ml after reconstitution, or a concentration of 150 U/ml. The pharmacy of the University Hospital Basel will distribute the investigational product to study sites in Switzerland. The Sponsor-Investigator will organize the distribution of the drug to non-Swiss sites in collaboration with the drug manufacturer Pharming Technologies B.V.

8.1.4 Storage Conditions

Conestat alfa will be stored at 2-25ºC at the pharmacy of the study sites (shelf life 48 months when stored at 2-25°C) in a dedicated, secure and temperature-monitored study room which will be inaccessible to unauthorized personnel. Drug accountability will be monitored according to ICH-GCP E6 (R2) Guideline for Good Clinical Practice. More details are described in the Study Pharmacy Manual.

8.2 Administration of experimental and control interventions

8.2.1 Experimental Intervention

Conestat alfa (Ruconest®) will be administered as slow (5 min for 4200 U dose and 10 min for 8400 U dose) intravenous injection via a peripheral or central intravenous line. A uniform dose of 8400 U (initial dose) and 4200 U (subsequent doses) was chosen irrespective of body weight. The licensed dosage for conestat alfa is weight-based (50 U/kg up to 84 kg and 4200 U for a bodyweight of $\geq$84 kg). This is based on the aim to at least achieve a level of 0.7 U/ml C1INH in patients with hereditary angioedema (lower limit of normal of C1INH activity). Simulation studies have revealed that this aim is achievable with the licensed dosing[48]. However, these simulations have also shown that C1INH levels will be lower

when using 50 U/kg compared to 4200 U in patients with a bodyweight < 84kg. As for the current trial the aim is not to correct underlying *absolute* C1INH deficiency (as in case of hereditary angioedema), but to ensure that a level of at least twice the serum concentration will be achieved in the vast majority of patients, we will use a fixed dose for all patients irrespective of body weight.

In patients with normal C1INH levels, the chosen dose will increase plasma C1-inhibitor activities by at least 100% (4200 U) and 200% (8400 U), respectively. To maximise efficacy conestat alfa will be administered repeatedly over 72 hours. Maximal volume of the injection is 28ml (4200 U) and 56ml (8400 U) per administration, respectively.

Repeated administration of conestat alfa was chosen for several reasons. First, hyperinflammation caused by SARS-CoV-2 is a phenomenon that may last for several days, and hence sustained inhibition of the CS and the KK system is required. Second, the elimination half-life of conestat alfa was determined at 2.5 hours[34]. Third a decline of C1 inhibitor activity to pre-administration levels was demonstrated within four to six hours after administration of conestat alfa at a dose of 50 U/kg[49]. Hence, in order to generate supra-physiological C1INH for the majority of time during a 72h interval, administration of a loading dose followed by repeated injections of conestat alfa was chosen.

Duration of exposure to Conestat alfa will be approximately 74 hours (up to 10 hours after the last dose administered 64 hours after the first dose). Participants will be followed in hospital for at least 12 hours after the last dose and via structured telephone interviews four weeks later.

8.2.2 Control Intervention

Participants in the control arm will receive SOC treatment according to local guidelines.

8.3 Dose / Device modifications

The only modification will be omission of subsequent doses of conestat alfa if the participant develops a type 1 allergic reaction including an anaphylactic reaction after any dose of conestat alfa. Other dose modifications are not expected, in particular will conestat alfa not ceased if the patient deteriorates already during the conestat alfa treatment interval. If the patient is discharged or transferred to another facility the intervention will be ceased.

8.4 Compliance with study intervention

Conestat alfa syringes for all administration time points on a given day will be prepared by a regular ward nurse. The study drug including the exact time for administration of the first and subsequent syringes will be entered into the electronic prescribing system/chart or added to the paper chart. As this study involves IV administration of the IMP by nurses, patient compliance measures are not necessary. IMP will be administered by a nurse according to the site's standard procedures. Two nurses will check the IMP with respect to dose, study number and patient's identity before administration to the patient. Nurses will document the syringe number and the time of administration in the electronic or paper chart.

8.5 Data Collection and Follow-up for withdrawn participants

Patients can leave the study at any time for any reason if they wish to do so without any consequences. The Investigator can decide to withdraw a patient from the study for medical reasons. A patient who experiences a treatment-emergent anaphylactic reaction will not be permitted to receive further treatment with conestat alfa.

Patients who withdraw informed consent will not be followed up. Although incomplete, data and samples collected up to the discontinuation will be analyzed. Personal data, samples and the confidentiality of these patients will be managed according to the same standards as per other evaluated completed patients.

Patients who are withdrawn from the study because they did not receive any study medication will not be followed up.

Patients who are discontinued from the study because of a type I allergic reaction during the treatment phase are followed in the same fashion as planned for all participants.

8.6 Trial specific preventive measures

All participants will receive standard supportive care established at the sites which may include off-label use of certain drugs that are deemed to be effective in COVID-19 (e.g. hydroxychloroquine,

lopinavir/ritonavir). In addition, escalation of treatment with off-label use of certain drugs (e.g. tocilizumab) in deteriorating participants is allowed during the trial. In terms of risk, conestat alfa, a recombinant human protein, has a very favourable side effect profile and major adverse events attributable to study medication are not expected in this study. Anaphylactic reactions are exceptionally rare and have only been observed in a single patient with a pre-existent allergy to rabbits. Although patients with rabbit allergy will be excluded from participating in the study, included subjects will be monitored for anaphylactic events during and at least for at least 12 hours after the last administration of conestat alfa. If patients develop type 1 allergic reactions following the administration of conestat alfa treatment according to in-house guidelines (potentially involving administration of antihistamines, corticosteroids, intravenous fluids and if needed adrenaline) will be provided.

No influence of conestat alfa on laboratory parameters or vital signs, including ECG was observed in previous clinical studies. Hence, we will not perform additional laboratory monitoring exceeding routine laboratory controls planned by the treating team (with the exception of the above mentioned additional biomarker analyses). No interactions with other drugs are known.

If female, patients must be/have: Post-menopausal, defined as the absence of menses for at least one year, OR Surgically sterile, defined as a bilateral tubal ligation at least 6 months prior to administration of study drug, bilateral oophorectomy, or complete hysterectomy, OR using an effective means of contraception with a failure rate less than 1% per year (e.g. oral contraceptives, contraceptive injections, intrauterine device, double-barrier method, male partner sterilization) OR a negative pregnancy test before study entry/randomisation. We expect the rate of female patients of childbearing age who may become pregnant to be <10% of all patients potentially eligible for this study. Due to the short treatment period (3 days) and half-life of conestat alfa (2.5 hours) an effective means of contraception is not mandatory after/during the treatment phase.

8.7 Concomitant Interventions (treatments)

All participants will receive standard supportive care established at the sites which may include off-label use of certain drugs that are deemed to be effective in COVID-19 (e.g. hydroxychloroquine, lopinavir/ritonavir, prophylactic anticoagulation). In addition, escalation of treatment with off-label use of certain drugs (e.g. tocilizumab) in deteriorating participants is allowed during the trial. Randomisation will be stratified by site to compensate for different standards of supportive care. Off-label use of drugs for the treatment of COVID-19 will be recorded in the eCRF. Escalation of treatment is one outcome of interest.

Patients are allowed to use all their regular medication during the trial. In addition, patients will receive all drugs required for the treatment of COVID-19 or its consequences. The care of the participant will remain unaffected by inclusion into the study or by study group assignment.

8.8 Study Drug / Medical Device Accountability

The in-house Pharmacy will be responsible for maintaining accurate drug storage and dispensing logs including records about shipments to the trial sites, return to the Sponsor and document on-site destruction if applicable. Drugs will be stored at each study site in accordance with GCP and GMP requirements and will be inaccessible to unauthorized personnel. Lot/batch numbers will be recorded. All unused and used conestat alfa vials will be retained at the site until drug inventory has been completed by the Monitor.

The ward nurses will use study drugs only within the framework of this clinical study and in accordance with this protocol. Receipt, distribution, return, and destruction (if any) of study drugs will be properly documented. Accountability records will include dates, quantities, batch/serial numbers, expiration dates, and patient numbers and sign off by the pharmacist or nurse. These records will adequately document that the patients were provided the doses as specified in the protocol and should allow reconciliation activities from Monitor for all conestat alfa received from the Sponsor.

The drug accountability process will be described in further detail separately in the Monitoring Manual.

8.9 Return or Destruction of Study Drug / Medical Device

Any study product that remains unused at the termination of the study will be returned to the Sponsor (Basel) or destroyed in accordance with institutional policies and this process will be recorded.

9. STUDY ASSESSMENTS

9.1 Study flow chart(s) / table of study procedures and assessments

Study Periods	Screening	Intervention Period				Follow-up	
Visit	1	2	3	4	5	6	7
Time (weeks or days, or hours)	d-1 to d0[1]	d0	d1	d2	d3-13 during admission*	d14 (+/-2d) or discharge	wk4 (+/-5d
In- /Exclusion Criteria	x						
Patient Information and Informed Consent	x						
Randomisation	x						
Demographics		x					
Medical History and Physical Examination		x				x	
Vital Signs including Respiratory Rate[9]		x	x	x	x	x	
Laboratory Tests (blood)		x	x		x[3]	x	
Virology testing		x[2]			x[2]	x[2]	
Administer Study Medication[4]		x	x	x	x		
Assessment of WHO ordinal scale		x	x	x	x	x	x
Assessment of ICU admission and/or mechanical ventilation		x	x	x	x	x	x
Laboratory assessment in detail:							
Routine full blood count		x[5]	x		x[3]	x	
Routine coagulation studies		x[5]	x		x[3]	x	
Routine biochemistry including LDH		x[5]	x		x[3]	x	
Serum pregnancy test	x[6]						
Ferritin, IL-6, D-Dimer		x[5]	x[11]		x[3]	x	
C1INH concentration[7]		x	x			x	
Additional samples in Basel[10]:		x					
Complement proteins (e.g. C4d, C5a)		x	x		x[3]	x	
Inflammatory cytokines (e.g. IL-10)		x	x		x[3]	x	
Contact activation and coagulation proteins (e.g. factor XII)		x	x		x[3]		
C1INH concentration and activity		x[8]	x[8]		x (d3, 7, 10)	x	
Urinary markers of renal injury		x	x		x[3]	x	
Blood volume (ml), total/study specific (Basel[10])	5/5 (5)	12/12 (22)	12/12 (22)		12/5 (10)	12/12 (17)	
Urine volume (ml), study specific in Basel[10]		5	5		5	5	
(Serious) Adverse Events		x	x	x	x	x	x

[1]Eligibility criteria have to be assessed within 72 hours after hospital admission. Randomisation may occur on the day of hospital admission.

[2]Monitoring of SARS-CoV-2 viral load will be performed in Basel and optional in other centers. This will include performance of a quantitative SARS-CoV-2 PCR from upper or lower respiratory tract at baseline

and subsequently once between day 4-7 (according to local standard) and again on day 14 if participants are still admitted or between day 10-13 if discharged earlier. Standard operating procedures include the performance of a second swab following a first negative swab.

[3]Routine laboratory tests will be performed on days 3, 5, 7, and 10 during hospital admission according to standard procedures of the study site, but will include a set of hematology, coagulation and biochemistry parameters. Additional blood samples will be collected at the same time.

[4]IMP will be administered during a 64 hour period (+/-4 hours). Trial medication will be stopped in participants that are discharged or transferred to another facility.

[5]Can be omitted if results are available from the same day.

[6]A pregnancy test will be performed in women who may become pregnant, are sexually active, and do not use adequate contraceptive measures.

[7]Collection of samples will be before 1st administration of study medication and subsequently at least 4 hours after the administration of study medication.

[8]Collection of samples will be before 1st administration, 10 (+/-5) minutes after the 1st administration (day 0) and before and 10 (+/-5) minutes after one administration on day 1.

[9]The worst value recorded on a given day will be documented.

[10]optional for other centers depending on their ability to collect, centrifuge, aliquot and store blood and urine samples at -80°C.

[11]IL-6 will not be measured on day 1

* If the patient was already discharged before day 7, the assessment of the WHO ordinal scale will be conducted via a short telephone interview (Score 1 or 2).

9.2 Assessments of outcomes

9.2.1 Assessment of primary outcome

For the analysis of the primary endpoint, illness severity will be assessed on each day after enrolment (worst status) with the use of the WHO Ordinal Scale for Clinical Improvement (score 0 will be omitted and score 6 and 7 will be combined) and the score on day 7 will be analysed stratified by its baseline value.

Patient State	Descriptor	Score
Uninfected	No clinical or virological evidence of infection	0
Ambulatory	No limitation of activities	1
	Limitation of activities	2
Hospitalized Mild disease	Hospitalized, no oxygen therapy	3
	Oxygen by mask or nasal prongs	4
Hospitalized Severe Disease	Non-invasive ventilation or high-flow oxygen	5
	Intubation and mechanical ventilation	6
	Ventilation + additional organ support – pressors, RRT, ECMO	7
Dead	Death	8

Figure 4: WHO ordinal scale for clinical improvement. For the current study, score 0 will be omitted, and score 6 and 7 will be combined.

The worst score on a given day (between 0:00 and 24:00 hours) will be used for the categorization

Patient State	Description	Score

Outpatient	No limitation in activities	1
	Limitation in activities	2
Hospitalized<br>Mild disease	No oxygen therapy	3
	Oxygen by mask or nasal prongs	4
Hospitalized<br>Severe disease	Non-invasive ventilation or high-flow oxygen	5
	Intubation, mechanical ventilation +/- additional organ support	6
Death	Death	7

9.2.2 Assessment of secondary outcomes

- For the analysis of time to clinical improvement of at least 2 points in the WHO Ordinal Scale for Clinical Improvement, clinical severity will be assessed every day until day 14 according to the Scale with the worst status for that day recorded
- For the analysis of the proportion of patients alive and not having required invasive or non-invasive ventilation at 14 days after enrolment, admission to ICU with invasive or non-invasive ventilation or death will be assessed every day until day 14.
- For the analysis of the proportion of patients with ALI within 14 days after enrolment, PaO_2/FiO_2 will be determined daily until day 14. This is only relevant for patients with arterial blood gas sampling performed in the ICU or rarely on the ward.

9.2.3 Assessment of other outcomes of interest

- The following assessments will be performed during the study: changes in WHO ordinal scale, ICU admission/discharge date, mechanical ventilation start/end date, admission/discharge date, all-cause mortality at 4 weeks
- The receipt of additional anti-inflammatory treatments such as tocilizumab will be recorded within 14 days
- Vital signs will be recorded during 14 days until discharge to determine the time to defervesence and clinical improvement
- For the analysis of virological clearance of SARS-CoV-2, samples from the upper or lower respiratory tract will be collected at baseline (if not already performed on admission) to confirm SARS-CoV-2 infection and again on day 4-7 and day 14/discharge (only when discharged on day 10-13). Subsequently, samples will be analysed at the central laboratory of the study site using a quantitative assay according to validated standard in-house procedures. A negative result will be confirmed by a second test at least 12 hours apart.
- For the analysis in changes of biomarker levels CRP- LDH, D-dimer, IL-6 and lymphocytes will be measured in blood samples collected on day 0, 1-3 and every second day afterwards until day 14 or discharge. Blood samples will be sent to and analysed by validated methods in the central laboratory of respective study sites according to standard in-house procedures
- The incidence of acute kidney injury will be evaluated according to the KDIGO classification. Baseline creatinine will be defined as a concentration obtained within a 12-month period before admission and considered to be representative of the baseline kidney function according to the judgment of the treating physician. If this reference value is not available, the minimum of either serum creatinine at the time of admission or study inclusion, or in patients without chronic kidney disease, a calculated serum creatinine concentration using the MDRD equation will be used.
- For the analysis of inflammatory proteins, parameters of the contact activation and coagulation system and cytokines, blood samples will be collected on day 0, 1, 3, 5, 7, 10 and day 14 or discharge (in Basel, optional for other centers). Blood samples will be sent to the central laboratory of the University Hospital Basel and the other centers, respectively, centrifuged, aliquoted and stored at -80°C. Subsequently, samples will be analysed in duplicate in batches by commercially available, validated ELISAs in the Clinical Immunology Laboratory (Prof. M. Trendelenburg) and the Immunology division of the central laboratory by

a blinded technician. Blank and zero sample and three quality control samples (low, medium and high concentration) in duplicate will be included in each run. Accuracy values of the quality control samples should be within +/- 15% of the nominal values (or study samples will be re-analysed). In addition, all samples from the same participant will be analysed together in the same analytical run.

- For the analysis of conestat alfa pharmacokinetics (C1INH activity and concentration), blood samples will be collected at baseline, 5-10 minutes after the first administration and again immediately before and 5-10 minutes after a subsequent administration on day 1 (in Basel, optional for other centers). Blood samples will be sent to and analysed by validated methods in the central laboratory of the University Hospital Basel according to standard in-house procedures. Samples from other centers will be stored at -80°C before being shipped to and analysed in Basel.
- For the analysis of renal injury markers, urine samples will be collected at baseline, day 1, 3, 5, 7, 10 and day 14 or discharge (in Basel, optional for other centers). Samples will be centrifuged, aliquoted and stored at -80°C. Subsequently, all samples will be analysed in duplicate in batched by commercially available, validated ELISAs in the Clinical Immunology Laboratory (Prof. M. Trendelenburg) in Basel.

9.2.4 Assessment of safety outcomes

9.2.4.1 Adverse events

Participants will be followed for adverse events during 4 weeks after enrolment. After the end of clinical follow-up there is no requirement to actively collect AEs including death.
Adverse events and clinical course including death within 4 weeks will be assessed by a structured telephone interview after 4 weeks. If necessary, medical charts will be reviewed, discharge summaries will be or the patients' general practitioner will be contacted regarding specific follow-up information. Every effort will be made to contact the trial subject and obtain complete data for follow-up. Conditions that started before signing informed consent will be recorded as medical history. Conditions that started or deteriorated after signing informed consent will be documented as AE. Definition of AE and SAE will be found in Section 10.
All AEs will be assessed and documented by the investigators according to seriousness, intensity, causal relationship with study treatment, action taken with study treatment (e.g. withdrawal), specific treatment for AE and outcome. In addition, time of onset and AE duration will be recorded. Procedures are defined in Section 10. AEs will be recorded on the respective eCRF pages.
Participants will be asked about any new events/complaints during the admission and at 4 weeks.
In addition, participants are asked to report any new events including hospital admission to the study team.

9.2.4.2 Laboratory parameters

Previous studies of conestat alfa have not shown any influence of conestat alfa on laboratory parameters. Hence, we will not perform additional laboratory monitoring exceeding routine laboratory controls planned by the treating team. If laboratory findings reveal abnormal values, they will be documented and reported as an AE if deemed clinical significant by the treating team or the investigators.

9.2.4.3 Vital signs

No study specific vital signs will be assessed. All patients will be monitored regarding blood pressure, heart rate, respiratory rate and oxygen saturation as per standard operating procedures for the treatment of COVID-19 patients.

9.2.5 Assessments in participants who prematurely stop the study

Patients who are withdrawn from the study will remain in the trial for the purpose of follow-up and data analysis and will be followed as planned with the following exceptions.

- Patients, who withdraw consent for all study measures, will be asked to have assessments performed as appropriate for the final 4 week study visit (i.e. a structured telephone interview). The same approach applies for all active study participants if the trial is stopped early. Patients who withdraw consent are at liberty to refuse any or all individual components of the final assessment.

- Patients who are withdrawn because they have not received any study medication will not be followed up and will not receive a final study assessment.

Patients who discontinue the study because of safety concerns will be followed as planned. Patient with a type I allergic reaction will be referred to our Allergy department for further work-up.

9.3 Procedures at each visit

9.3.1 Split into subtitles by type of visit

Visit 1, Screening (Day -1 or day 0):

Informed consent will be obtained before any study-specific tests or evaluations will be performed.

- Patients who are admitted to the medical ward with confirmed or suspected SARS-CoV-2 infection will be screened within 72 hours after admission on the ward. Screening will be based on routinely available data and laboratory tests with only the pregnancy test as study-specific intervention/test if necessary before screening of the patients. Results from prior tests that will be used for screening potentially eligible subjects will be recorded in the eCRF.
- Selection of potential participants according to inclusion and exclusion criteria.
- Explanation of patient information including study purpose and signature of informed consent form.
- Study enrolment
- Assignment of the trial subject identification number
- Pregnancy test if applicable.
- Eligibility check (in- and exclusion criteria)
- Randomisation, which can either occur on the same day of hospital admission or within 72 hours after hospital admission.

Visit 2, Intervention period (Day 0)

- Assessment of demographics, medical history and concomitant medication
- Assessment of vital signs including respiratory rate.
- Assessment of WHO ordinal scale and need for oxygen supplementation
- Collection of blood (12ml) for analysis of ferritin, Il-6, D-Dimer, C1INH concentration, and in Basel (optional for other centers): collection of blood (5ml) and urine (5ml) for the analysis of complement proteins, parameters of the contact activation and coagulation system, renal injury markers, and inflammatory cytokines. Routine full blood count, coagulation and biochemistry studies will only be performed if results from prior tests on the same day are not available.
- Assessment of reference serum creatinine
- Virology testing (quantitative SARS-CoV-2 PCR) on a nasopharyngeal sample if not already performed before inclusion into the study. This is optional for other centers except Basel.
- Following randomisation, participants in the active treatment group will receive 8400 U of conestat alfa as slow intravenous injection. The time of dosing and the dose will be recorded. Subsequently, 4200 U will be administered every 8 hours in the active trial group.
- AEs occurring after the administration will be recorded.
- Only in Basel and in the active treatment group: Collection of blood (5ml) for the analysis of C1INH levels and activity 10 (+/-5 minutes) after the first administration.

Visit 3, Intervention period (Day 1)

- Administration of 4200 U of conestat alfa every 8 hours in the active treatment group as per treatment schedule. The time of dosing and the dose will be recorded.
- Assessment of vital signs including respiratory rate.
- Assessment of WHO ordinal scale and need for oxygen supplementation
- Documentation of ICU admission and/or non-invasive or invasive ventilation
- Collection of blood (12ml) for analysis of ferritin, D-Dimer, C1INH concentration, and in Basel (optional for other centers): collection of blood (5ml) and urine (5ml) for the analysis of complement proteins, parameters of the contact activation and coagulation system, renal injury markers and inflammatory cytokines. Routine full blood count, coagulation and biochemistry studies will only be performed if results from prior tests on the same day are not available.
- Only in Basel and in the active treatment group: Collection of blood (5ml) for analysis of C1INH levels and activity 10 (+/-5 minutes) after any of the three administrations of conestat alfa on that day.

- Assessment of AEs.

Visit 4, Intervention period (Day 2)

- Administration of 4200 U of conestat alfa every 8 hours in the active treatment group as per treatment schedule. The time of dosing and the dose will be recorded.
- Assessment of vital signs including respiratory rate.
- Assessment of WHO ordinal scale and need for oxygen supplementation
- Documentation of ICU admission and/or non-invasive or invasive ventilation
- Assessment of AEs.

Visit 5, Intervention period (Day 3-13 during hospital admission)

- Completion of treatment with conestat alfa (9 administrations in total including the first 8400 U administration) in the active treatment group as per treatment schedule. The time of dosing and the dose will be recorded.
- Assessment of vital signs including respiratory rate.
- Assessment of WHO ordinal scale and need for oxygen supplementation. If the patient was already discharged before day 7, the assessment of the WHO ordinal scale will be conducted via a short telephone interview (Score 1 or 2).
- Documentation of ICU admission and/or non-invasive or invasive ventilation
- Routine laboratory tests will be performed on days 3, 5, 7 and 10 during admission according to standard procedures, and should include a set of hematology, coagulation and biochemistry parameters. Additionally, blood (5ml) for analysis of ferritin, IL-6, D-Dimer, C1INH concentration will be collected at the same time point, and in Basel (optional for other centers): collection of blood (5ml) and urine (5ml) for the analysis of complement proteins, parameters of the contact activation and coagulation system, renal injury markers and inflammatory cytokines.
- Virological assessment (quantitative SARS-CoV-2 PCR in respiratory specimen) should be performed on day 4-7 (according to local standard). This is optional for all centers except Basel.
- Assessment of AEs

Visit 6, Follow-up (Day 14 (+/-2 days) or at discharge)

- Physical examination including body weight.
- Assessment of vital signs including respiratory rate.
- Assessment of WHO ordinal scale and need for oxygen supplementation
- Documentation of ICU admission and/or non-invasive or invasive ventilation
- Laboratory assessment including routine hematology, coagulation and biochemistry studies (7ml). Routine laboratory tests will be performed every second day during admission according to standard procedures. Additional sample collection will be required in patients without routine testing on the day of discharge. Additionally, blood (5ml) for analysis of ferritin, IL-6, D-Dimer, C1INH concentration will be collected at the same time point, and in Basel (optional for other centers): collection of blood (5ml) and urine (5ml) for the analysis of complement proteins, renal injury markers and inflammatory cytokines.
- Virological assessment (quantitative SARS-CoV-2 PCR in respiratory specimen) will be performed on day 14 (when still admitted) or at discharge if on day 10-13. This is optional for all centers except Basel.
- Assessment of AEs.

Visit 7, Follow-up (week 4 +/- 5 days, telephone interview or study visit if still admitted)

- Assessment of adverse events, WHO ordinal scale, admission to ICU and/or mechanical ventilation by structured telephone interview. If necessary, medical charts will be reviewed, discharge summaries will be obtained or the patients' general practitioner will be contacted regarding specific follow-up information.

10. SAFETY

10.1 Drug studies

The Sponsor's SOPs provide more detail on safety reporting.

During the entire duration of the study, all adverse events (AE) and all serious adverse events (SAEs) are collected, fully investigated and documented in source documents and electronic case report forms (eCRF). Study duration encompassed the time from when the participant signs the informed consent until the last protocol-specific procedure has been completed, including a safety follow-up period.

As the frequency of comorbidities in the patient population under study is significant and clinical complications are common in patients with COVID-19 infection, serious adverse events are possible in this setting. Hence, we will establish periodic interim safety review meetings by an independent committee (see also Chapter 1.6).

10.1.1 Definition and assessment of (serious) adverse events and other safety related events

An **Adverse Event (AE)** is any untoward medical occurrence in a patient or a clinical investigation participant administered a pharmaceutical product and which does not necessarily have a causal relationship with the study procedure. An AE can therefore be any unfavourable and unintended sign (including an abnormal laboratory finding), symptom, or disease temporally associated with the use of a medicinal (investigational) product, whether or not related to the medicinal (investigational) product. [ICH E6 1.2]. Any medical conditions present before signing the informed consent will be reported in the medical history. Any AEs that occur between screening and first treatment are considered as Non-Treatment Emergent Adverse Events (TEAE).

A **Serious Adverse Event (SAE)** is classified as any untoward medical occurrence that:

- results in death,
- is life-threatening,
- requires in-patient hospitalization or prolongation of existing hospitalisation,
- results in persistent or significant disability/incapacity, or
- is a congenital anomaly/birth defect.

In addition, important medical events that may not be immediately life-threatening or result in death, or require hospitalisation, but may jeopardise the patient or may require intervention to prevent one of the other outcomes listed above should also usually be considered serious. [ICH E2A]. An elective or pre-planned hospital admission will not be considered as a SAE. Note: an AE or adverse drug reaction is considered 'life-threatening' if, in view of either the Investigator or Sponsor, its occurrence places the patient at immediate risk of death. It does not include an event that, had it occurred in a more severe form, might have caused death.

SAEs should be followed until resolution or stabilisation. Participants with ongoing SAEs at study termination (including safety visit) will be further followed up until recovery or until stabilisation of the disease after termination.

Regardless of the assumed causal relationship, all AE volunteered by the trial subjects or observed by the investigator and/or his staff must be recorded on the AE forms. Records of AEs include patient data, description of the event, intensity, severity, relationship to the study drugs, temporal relation to the study drugs, action taken, description of outcome and documentation of the results of diagnostic and therapeutic measures.

Assessment of Causality

Both Investigator and Sponsor-investigator make a causality assessment of the event to the study drug, based on the criteria listed in the ICH E2A guidelines:

Relationship	Description
Definitely	Temporal relationship<br>Improvement after dechallenge*<br>Recurrence after rechallenge<br>(or other proof of drug cause)

Probably	Temporal relationship<br>Improvement after dechallenge<br>No other cause evident
Possibly	Temporal relationship<br>Other cause possible
Unlikely	Any assessable reaction that does not fulfil the above conditions
Not related	Causal relationship can be ruled out
*Improvement after dechallenge only taken into consideration, if applicable to reaction	

Unexpected Adverse Drug Reaction

An "unexpected" adverse drug reaction is an adverse reaction, the nature or severity of which is not consistent with the applicable product information (e.g. Investigator's Brochure for drugs that are not yet approved and Product Information for approved drugs, respectively). [ICH E2A]

Suspected Unexpected Serious Adverse Reactions (SUSARs)

The Sponsor-Investigator evaluates any SAE that has been reported regarding seriousness, causality and expectedness. If the event is related to the investigational product and is both serious and unexpected, it is classified as a SUSAR.

Assessment of Severity

The Investigator will be required to assess the intensity of the AE and to record this assessment in the source documents. It is important to distinguish between serious criteria and severity of an AE. Severity is a measure of intensity whereas seriousness is determined by the criteria mentioned above. Investigators will use the following categories to quantify severity.

- Mild/grade 1: events require minimal or no treatment and do not interfere with the patient's daily activities.
- Moderate/grade 2: events result in a low level of inconvenience or concern with the therapeutic measures. Moderate events may cause some interference with functioning.
- Severe/grade 3: events interrupt a patient's usual daily activity and may require systemic drug therapy or other treatment. Severe adverse events are usually incapacitating.
- Life threatening/grade 4: potentially life-threatening or disabling. High-risk medical interventions.
- Fatal/grade 5: patient died.

Changes in the severity of an AE will be documented to allow an assessment of the duration of the event at each level of intensity to be performed. AEs characterized as intermittent require documentation of onset and duration of each episode.

10.1.2 Reporting of serious adverse events (SAE) and other safety related events

Reporting of SAEs

The Sponsor-Investigator is responsible for informing all competent authorities (CA) and ethical commitees (CEC) of the concerned Member States of any SAEs as per local requirements. Reporting will be performed according to local regulations. This may include the following:

All SAEs must be reported immediately and within a maximum of 24 hours to the Sponsor-Investigator of the study. The Sponsor-Investigator will re-evaluate the SAE and return the form to the site.

SAEs resulting in death are reported to the Ethics Committee (for Swiss sites via BASEC) within 7 days. SAEs resulting in death will be reported to Pharming Technologies, B.V., the manufacturer of the IMP according to the same timelines.

The other in the trial involved Ethics Committees receive SAEs resulting in death in Switzerland via Sponsor-Investigator (for Swiss sites via BASEC) within 7 days.

Reporting of SUSARs

The Sponsor-Investigator will report all SUSARs in compliance with applicable laws and regulations to all Competent Authorities of the concerned Member States (e.g. Swissmedic in Switzerland, AIFA in Italy) and CECs of all study sites (lead EC in Switzerland) within 7 days, if the event is fatal or life-threating or within 15 days (all other events).

The Sponsor-Investigator must inform all Investigators participating in the clinical study of the occurrence of a SUSAR. All in the trial involved Ethics Committees will be informed about SUSARs in Switzerland via Sponsor-Investigator via BASEC according to the same timelines and according to local regulations in other countries

In addition, the SUSARs will be provided to Pharming Biotechnologies BV, the manufacturer of the IMP, within one business day of submission to the Competent Authorities using a format that is accepted by Pharming Biotechnologies, B.V.

Reporting of Safety Signals

All suspected new risks and relevant new aspects of known adverse reactions that require safety-related measures, i.e. so called safety signals, must be reported to the Sponsor-Investigator within 24 hours. The Sponsor-Investigator must report the safety signals within 7 days to the Ethics Committee (local event via local Investigator) (for Swiss sites via BASEC) and to Swissmedic in case of a category B or C study and according to local regulations in other countries

The Sponsor-Investigator must immediately inform all participating Investigators about all safety signals. The other in the trial involved Ethics Committees will be informed about safety signals in Switzerland via the Sponsor-Investigator and according to local regulations in other countries.

Reporting and Handling of Pregnancies

Pregnancy is an exclusion criteria for the study. Women of childbearing potential must have a negative pregnancy test to be eligible for the study. A pregnancy test will be performed in women who may become pregnant. Pregnant participants must immediately be withdrawn from the clinical study. It is highly unlikely for a pregnancy to occur during the treatment phase. Any pregnancy during the treatment phase of the study and within 30 days after discontinuation of study medication will be reported to the Sponsor-Investigator within 24 hours. The course and outcome of the pregnancy should be followed up carefully, and any abnormal outcome regarding the mother or the child should be documented and reported.

Periodic reporting of safety

The Sponsor Investigator will submit an Annual Safety Report once a year to the CAs and CES of all study sites (lead CEC in Switzerland) or more often if required by local regulations. The Annual Safety Report contains information from all site including information from sites outside of Switzerland. The Sponsor-Investigator prepares the report. In addition, a copy of the report will be sent to Pharming Biotechnologies BV.

10.1.3 Follow up of (Serious) Adverse Events

Participants with SAEs (whether or not related to the study drug) will be monitored until resolution or until the event is considered chronic and/or stable by the investigator and/or other physician who has the responsibility for the subject's medical care. Treating physicians including general practitioners will be contacted for follow-up results. If necessary, participants will be contacted directly for follow-up. Follow-up SAE reports will be reported according to the same timelines as initial reports, as soon as new significant information becomes available. The outcome of AEs will be documented.

11. STATISTICAL METHODS

11.1 Hypothesis

Null hypothesis: Early administration of conestat alfa in addition to SOC is not associated with an improved clinical outcome in admitted COVID-19 patients as reflected by a lower score on the WHO Ordinal Scale on day 7 compared to SOC alone.

Alternative hypothesis: Early administration of conestat alfa in addition to SOC is associated with an improved clinical outcome in admitted COVID-19 patients as reflected by a lower score on the WHO Ordinal Scale on day 7 compared to SOC alone .

11.2 Determination of Sample Size

This is a pilot study investigating the efficacy and safety of conestat alfa in the prevention of clinical deterioration in a high-risk population with COVID-19.

There is no reliable information available on the clinical evolution of the population included in the trial. Therefore a precise quantitative pre-definition of the sample size of a population needed to obtain clinically significant primary endpoint is not possible at this point in time.

The assumptions which are proposed and the criteria to monitor and assess the primary endpoint are as follows:

The primary endpoint is a 7-point scale and the standard deviation σ can be expected as 1.5 points. A relevant effect δ is an advantage of at least 1 point. Then, the standardized difference is about δ/σ = 0.67. For a fixed sample size design with a two-sided significance level of $\alpha = 0.05$ and a power of $1 - \beta = 0.80$, a sample size of N = 2 x 38 is necessary. For a 2:1-randomization, a nonparametric analysis by the stratified logrank-test, and an adaptive group sequential analysis, the overall sample size is estimated as 120 = 80 + 40.

Adaptive design:

Two interim analyses after 40 and 80 patients are planned according to the Pocock adjusted levels $\alpha_p = 0.0221$. Based on the results of an interim analysis the sample size can be adjusted (Lehmacher, Wassmer, 1999). There are no prespecified futility margins, but the DSMB can recommend stopping the study in the case of insufficient interim results.

The primary efficacy endpoint and the main secondary efficacy endpoints will be analyzed at latest at 14 days after start of treatment.

11.3 Statistical criteria of termination of trial

There are no prespecified futility margins, but the DSMB can recommend stopping the study in the case of insufficient interim results.

11.4 Planned Analyses

11.4.1 Datasets to be analysed, analysis populations

A review of the database will be conducted in a blinded manner shortly before the database will be locked, and any decisions made at that meeting (blinded Data Review Meeting) concerning the statistical analysis, e.g., additional outcomes and populations, pooling of sites for efficacy analysis, will be documented in the Statistical Analysis Plan (SAP).

Missing data for the primary endpoint will be imputed by means of the Last-observation-carried-forward (LOCF) method.

Safety Population: The Safety Population is defined as all patients who received at least one dose of conestat alfa. Statistical analyses will be based on the actual treatment the patient received.

Full Analysis Set/Intent-to-Treat Population: The FAS/ITT Population is defined as all patients who are randomly allocated to a study arm. Statistical analyses will be based on the treatment arm to which the patient was allocated.

For quantitative data, the number of patients with non-missing information, means, medians, standard

deviations and extremes will be determined. For qualitative data, absolute and relative frequencies will be calculated. Moreover, changes from baseline (V2, d0) will be calculated.

Per Protocol Population: The PP Population is defined as all patients in the FAS/ITT Population who complete the study and who do not have any major protocol violations, including the following:

- Patients who had major inclusion/exclusion criteria violations.

Sufficient compliance is expected because of IV administration of the IMP.

The PP Population will be determined by a blinded review in the Data Review Meeting (DRM) of the data prior to database lock.

The primary efficacy analysis will be based on the FAS/ITT Population in this superiority trial; an additional efficacy analysis will be performed on the PP Population. The Safety Population will be used for all safety analyses.

11.4.2 Primary Analysis

The primary endpoint WHO 7-point outcome scale at day 7 will be analyzed by nonparametric logrank test stratified by its baseline values with two-sided α-level of 5 %.

Two adaptive interim analyses after 40 and 80 patients are planned according to the Pocock adjusted levels $\alpha_p = 0.0221$. The results of the sequential groups are combined by the inverse-nomal-method (Lehmacher, Wassmer, 1999). There are no prespecified futility margins, but the DSMB can stop the study in the case of insufficient interim results.

Furthermore, 95% confidence intervals will be determined, and the results will be presented graphically by means of box-and-whisker plots.

The analyses will be carried out by the trial biostatistician.

11.4.3 Secondary Analyses

The secondary endpoints are time to improvement of at least 2 points on the WHO 7-point outcome scale, the proportion of subjects with an ALI (defined by PaO2/FiO2 ratio of <300mmHg) within 14 days after enrolment and the proportion of participants alive and not having required invasive or non-invasive ventilation at 14 days after enrolment. They will be tested only after a significant test of the primary endpoint (a priori ordered hypotheses), therefore, no alpha adjustment is necessary.

Quantitative secondary study parameters will be described based on their mean, standard deviation, median, IQR, minimums and maximums and portrayed by Kaplan-Meier plots and compared with the logrank test.

Qualitative secondary study parameters will be analyzed by means of absolute and relative frequencies. Pairwise Chi-Square tests will be carried out in order to compare each of the active treatments to placebo. Moreover, 95% confidence intervals for the treatment differences will be calculated.

Other outcomes of interest will be analyzed by means of descriptive and exploratory statistics.

The analyses will be carried out by the trial biostatistician.

11.4.4 Interim analyses

Two interim analyses after 40 and 80 patients are planned according to the Pocock adjusted levels $\alpha_p = 0.0221$. Based on the results of an interim analysis the sample size can be adjusted (Lehmacher, Wassmer, 1999). There are no prespecified futility margins, but the DSMB can recommend stopping the study in the case of insufficient interim results.

11.4.5 Safety analysis

The assessment of safety will be based mainly on the frequency of adverse events (AEs) that are treatment-emergent (TEAEs). Formal tests will not be conducted for differences in safety parameters between treatment groups.

The incidence of all treatment-emergent AEs will be tabulated after grouping by system organ class and preferred term. For each preferred term and summarized over each system organ class overall, the

table will present the absolute number and proportion (%) of patients in each treatment group in whom the event occurred. The incidence of all suspected-IMP-related AEs will be tabulated similarly. The incidence of all treatment-emergent AEs will also be tabulated by severity categories.

Safety will also be summarized with respect to vital signs as mean levels by visit and change from baseline. Abnormal observations on physical exams will be listed.

11.4.6 Deviation(s) from the original statistical plan

Deviations from the planned analyses will be discussed by the Sponsor-Investigator and the biostatistician and consequently justified and reported in the final reports and the publications.

11.5 Handling of missing data and drop-outs

Withdrawn/discontinued participants will not be replaced.

Missing data for the primary variable will be imputed by means of the Last-observation-carried forward (LOCF) method.

12. QUALITY ASSURANCE AND CONTROL

The Sponsor-Investigator and the local site investigators are responsible for proper training of all involved study personnel and for implementing and maintaining quality assurance and quality controls systems with written SOPs and working instructions. All SOPs and working instructions will be prepared and critically evaluated by the investigators and study nurses before commencement of the trial and by the monitoring organization.

12.1 Data handling and record keeping / archiving

12.1.1 Case Report Forms

Study data will be recorded in an electronic data capture (EDC) system via an electronic Case Report Form (eCRF) which is provided by the Clinical Trial Unit (CTU), University Hospital Basel (secuTrial®). A unique study code will be used for identification of participants in the eCRF. Subjects will not be identified on the eCRF by name or date of birth.

Only authorized personnel will be able to make eCRF entries via a personalized login to the eCRF and are responsible for entering complete data.

If source data is available as a print-out (e.g. laboratory values), this print-out will be kept on file in a source data folder, and the data necessary for the study is to be transferred to the eCRF immediately.

12.1.2 Specification of source documents

Source data include the paper and electronic records of the hospitals at the respective study sites and all study documents (AE/SAE form, informed consent forms, laboratory reports etc.). Source data includes demographic data, visit dates, informed consent forms, randomisation numbers, SAEs, AEs, concomitant medication, results of physical examination and information related to COVID-19 infection (e.g. data from viral sampling). Source data may be found either in the electronic or paper-based records of the hospitals at the respective study sites or as a print-out of study-related laboratory results which will be mailed to the local investigators from the central laboratory hospitals at the respective study sites or will be provided to the Sponsor-Investigator by the blinded technician of the Clinical Immunology Laboratory of the University Hospital Basel (only relevant for the subgroup of patients included in Basel).

12.1.3 Record keeping / archiving

All study data will be archived for a minimum of 10 years after study termination or premature termination of the clinical trial. Study data will be archived in a password protected database on a protected server at the University Hospital Basel. Blood samples for the subgroup of patients undergoing analysis of inflammatory proteins will be stored in a dedicated freezer with limited access at the Department of Biomedicine of the University Hospital Basel. Any study relevant source data and documents will be archived at each study site for a minimum of 10 years.

12.2 Data management

Study data will be captured via an online Clinical Data Management System (CDMS) secuTrial, based at the IT-department of the University Hospital Basel. The data collected is entered into the study eCRF. An audit trail will maintain a record of initial entries and any changes made; time and date of entry; and user name of person authorizing entry or change. For each patient enrolled an eCRF must be completed. The principal investigator and Co-Investigator at the study site will be responsible for assuring that the data entered into the eCRF is complete, accurate, and that the entry and updates are performed in timely manner. If a patient withdraws from the study, the reason must be noted on a dropout form of the eCRF.

12.2.1 Data Management System

The eCRF will be implemented by the Data management group at the Clincial Trial Unit (CTU) of the University Hospital Basel using the Clinical Data Management System (CDMS) secuTrial. The CDMS runs on a server maintained by the IT-department of the University Hospital Basel. Additional storage capacity can be added as needed. Data entry will be performed by trained clinical investigators.

12.2.2 Data security, access and back-up

The CDMS is accessible via a standard browser on devices with internet connection. Password

protection and user-right management ensures that only authorized study investigators, monitors, data managers and local authorities (if necessary) will have access to the data during and after the study. User administration and user training is performed by the CTU Basel according to predefined processes. An integrated audit trail system will maintain a record of initial entries and changes made; time and date of entry; and user name of person authorizing entry or change. Backup of secuTrial study data is performed regularly according to the processes of the IT-department of the University Hospital Basel.

12.2.3 Analysis and archiving

The CDMS will be locked after eCRF data entry is completed, all data has been monitored and raised queries have been resolved. The complete study dataset is exported from the database and transferred to the study statistician as well as the principal investigator through a secure channel. The statistical analysis will be performed completely independent by the involved statistician of the CTU Basel. The exported data will be archived for 10 years by the principal investigator.

12.2.4 Electronic and central data validation

Data entered into the CDMS will be validated for completeness and discrepancies automatically. The data will be reviewed by the responsible investigator as well as an independent monitor (please refer to section 12.3.). The monitor will raise queries using the query management system implemented in secuTrial. Designated investigators have to respond to the query and confirm or correct the corresponding data. Thereafter the monitor can close the query.

12.3 Monitoring

Monitoring will be carried out according to a predefined monitoring plan by the Clinical Trial Unit, Department of Clinical Research, University Hospital Basel, which is independent from the investigators. Study documentation and all source data/documents will be accessible to monitors and all questions will be answered during inspections. Participants' data will be kept strictly confidential during the monitoring visits.

12.4 Audits and Inspections

Audits may be conducted by CEC and CA and are independent from investigators.

Study documentation and all source data/documents will be accessible to auditors/inspectors and all questions will be answered during inspections. Participants' data will be kept strictly confidential during the monitoring visits.

12.5 Confidentiality, Data Protection

All staff involved in the study is obliged to follow the ICH-GCP regulations, local requirements and privacy policy. Direct access to source documents will be permitted for purposes of monitoring (please refer to section 12.3.), audits and inspections (please refer to section 12.4).

Participants' personal/clinical data will be coded and entered into an online clinical trial database (SecuTrial®) which is a password-secured database provided by the Clincial Trial Unit Basel. Data will be archived. Any hard copies of the source documentation will be maintained in a locked filing cabinet with limited access.

Study data entered into the eCRF is only accessible by authorized persons. Once all data is entered into the CDMS and monitoring is completed, the database will be locked and closed for further data entry. The complete dataset is then exported and transferred to the study statistician as well as the principal investigator through a secure channel.(please refer to section 12.2.).

Subject confidentiality will be maintained throughout the clinical study in a way that ensures the information can always be tracked back to the source data. For this purpose, a unique subject identification code will be used that allows identification of all data reported for each subject.

Blood samples will be de-identified, labelled with a unique study code and stored in a dedicated freezer at the central laboratory of the University Hospital Basel until analysis and thereafter in a dedicated freezer at the Department of Biomedicine, University Hospital Basel. Samples will be destroyed 10 years after study termination.

Data and samples will be accessible only to authorized staff for scientific purposes. Throughout the study and during aforementioned inspections, strict confidentiality is guaranteed. In any publication

and/or presentation, information will be provided in such a way that the participants cannot be identified, except with their permission and individual patient data will not be shown.

Direct access to source documents will be permitted for the purpose of monitoring, audits and inspections by respective authorities.

12.6 Storage of biological material and related health data

Blood samples will be stored at -80°C in a dedicated freezer with limited access to unauthorized personal. The same applies to study data, which will be stored in a password secured database, located at the server of the USB and maintained by qualified IT specialists. All data including source data (patients' charts) are stored for ten years and will only be used for the present clinical trial. Blood samples will be stored until all planned analyses have been conducted (approximately 2-3 years after completion of the study). Data and samples will not be used for future clinical studies or projects.

13. PUBLICATION AND DISSEMINATION POLICY

Data derived from this clinical trial are considered the property of the Investigators of this trial.

Study results will be presented at national and international conferences and published in peer reviewed medical journals. Trial results will be disseminated to the public and patients through publications in national and international journals, presentations at conferences and through publication of results in a public registry.

As a multicenter study, the first publication is intended to be a multicenter publication of the results of the overall study. In these cases, the Sponsor-Investigator will be responsible for these activities and will work with the Investigators to determine how the manuscript is written and edited, the number and order of authors, the publishing journal/newsletter to which it will be submitted, and other related issues. Intended publications will be discussed among the investigators with the Sponsor-Investigator having the ultimate authority to decide the appropriate choice of medical journal(s). The contributions of any contributor to the project will be taken into account in a fair and collegial way. Persons qualify for authorship if they have contributed significantly to the trial. The last author of the main publication will be the Sponsor-Investigator, Michael Osthoff. If a contributor will not qualify for authorship, her/his contribution will be mentioned as an acknowledgment.

Any individual publications, presentations or other disclosures of any study results by the centers or the local investigator shall require the Sponsor-Investigator’s written consent and shall not occur until after the multicenter publication is published, which is intended to occur within 12 months after completion of the study at all study sites and lock of the database at all study sites.

Publications will by prepared without the use of professional writers. This may include sharing the full protocol and any participant-level data. The manuscript of the main publication will be prepared by the PI or a study member of the Division of Internal Medicine at the University Hospital Basel and sent to the other parties for comments and revision.

All proposed publications are to be provided to Pharming Biotechnology B.V. a minimum of 60 days before submission to prospectively review any proposed publication, abstract, other type of disclosure that reports the results of the study. Pharming Biotechnology B.V. may wish to disclose results of the study after publication/presentation.

All financial support will be disclosed in any publication of study results.

In accordance with national and local requirements, this study will be listed in a publicly accessible clinical studies registry and be given a unique identifier (e.g. ClinicalTrials.gov). Additionally, the results of this study will be disclosed on a publicly accessible clinical studies results database, regardless of the outcome.

14. FUNDING AND SUPPORT

14.1 Funding

The trial will be funded by

- a grant (4078P0_198403 / 1) from the Swiss National Science Foundation (NFP-78)
- an investigator initiated research grant including free IMP from Pharming Technologies B. V.
- departmental funds of the study sites
- the Wissenschaftspool Medizin, Universitätsspital Basel (for setup/management of the electronic database and monitoring of the study)

.

14.2 Other Support

The study will be supported by

- Clinical Immunology Laboratory, Department of Biomedicine, University Hospital Basel (Prof. Marten Trendelenburg): measurement of inflammatory proteins; storage of samples after analysis.
- Department of Laboratory Medicine, University Hospital Basel (Prof. Katharina Rentsch): measurement of blood samples, storage of samples until analysis.
- Clinical Trial Unit, University Hospital Basel: Monitoring of the study
- Pharmacy department, University Hospital Basel: storage of IMP
- local trial sites

15. INSURANCE

All trial subjects are covered for any injury which may occur as a result of the study participation by insurance provided by the institutions at the study site.

16. REFERENCES

1. van Doremalen N, Bushmaker T, Morris DH, Holbrook MG, Gamble A, Williamson BN, Tamin A, Harcourt JL, Thornburg NJ, Gerber SI, Lloyd-Smith JO, de Wit E and Munster VJ. Aerosol and Surface Stability of SARS-CoV-2 as Compared with SARS-CoV-1. *N Engl J Med*. 2020;382:1564-1567.

2. John Hopkins University & Medicine. Coronarvirus Resource Center. Coronarvirus COVID-19 Global Cases by the CSSE. Accessed on 28 April 2020.

3. Novel Coronavirus Pneumonia Emergency Response Epidemiology T. [The epidemiological characteristics of an outbreak of 2019 novel coronavirus diseases (COVID-19) in China]. *Zhonghua Liu Xing Bing Xue Za Zhi*. 2020;41:145-151.

4. Siddiqi HK and Mehra MR. COVID-19 Illness in Native and Immunosuppressed States: A Clinical-Therapeutic Staging Proposal. *J Heart Lung Transplant*. 2020.

5. Yang X, Yu Y, Xu J, Shu H, Xia J, Liu H, Wu Y, Zhang L, Yu Z, Fang M, Yu T, Wang Y, Pan S, Zou X, Yuan S and Shang Y. Clinical course and outcomes of critically ill patients with SARS-CoV-2 pneumonia in Wuhan, China: a single-centered, retrospective, observational study. *Lancet Respir Med*. 2020.

6. Qin C, Zhou L, Hu Z, Zhang S, Yang S, Tao Y, Xie C, Ma K, Shang K, Wang W and Tian DS. Dysregulation of immune response in patients with COVID-19 in Wuhan, China. *Clin Infect Dis*. 2020.

7. Wu C, Chen X, Cai Y, Xia J, Zhou X, Xu S, Huang H, Zhang L, Zhou X, Du C, Zhang Y, Song J, Wang S, Chao Y, Yang Z, Xu J, Zhou X, Chen D, Xiong W, Xu L, Zhou F, Jiang J, Bai C, Zheng J and Song Y. Risk Factors Associated With Acute Respiratory Distress Syndrome and Death in Patients With Coronavirus Disease 2019 Pneumonia in Wuhan, China. *JAMA Intern Med*. 2020.

8. Walport MJ. Complement. Second of two parts. *N Engl J Med*. 2001;344:1140-4.

9. Walport MJ. Complement. First of two parts. *N Engl J Med*. 2001;344:1058-66.

10. Thielens NM, Tacnet-Delorme P and Arlaud GJ. Interaction of C1q and mannan-binding lectin with viruses. *Immunobiology*. 2002;205:563-74.

11. Kase T, Suzuki Y, Kawai T, Sakamoto T, Ohtani K, Eda S, Maeda A, Okuno Y, Kurimura T and Wakamiya N. Human mannan-binding lectin inhibits the infection of influenza A virus without complement. *Immunology*. 1999;97:385-92.

12. Bibert S, Piret J, Quinodoz M, Collinet E, Zoete V, Michielin O, Menasria R, Meylan P, Bihl T, Erard V, Fellmann F, Rivolta C, Boivin G and Bochud PY. Herpes simplex encephalitis in adult patients with MASP-2 deficiency. *PLoS Pathog*. 2019;15:e1008168.

13. Schiela B, Bernklau S, Malekshahi Z, Deutschmann D, Koske I, Banki Z, Thielens NM, Wurzner R, Speth C, Weiss G, Stiasny K, Steinmann E and Stoiber H. Active Human Complement Reduces the Zika Virus Load via Formation of the Membrane-Attack Complex. *Frontiers in immunology*. 2018;9:2177.

14. Bermejo-Jambrina M, Eder J, Helgers LC, Hertoghs N, Nijmeijer BM, Stunnenberg M and Geijtenbeek TBH. C-Type Lectin Receptors in Antiviral Immunity and Viral Escape. *Frontiers in immunology*. 2018;9:590.

15. Gralinski LE, Sheahan TP, Morrison TE, Menachery VD, Jensen K, Leist SR, Whitmore A, Heise MT and Baric RS. Complement Activation Contributes to Severe Acute Respiratory Syndrome Coronavirus Pathogenesis. *mBio*. 2018;9.

16. Jiang Y, Zhao G, Song N, Li P, Chen Y, Guo Y, Li J, Du L, Jiang S, Guo R, Sun S and Zhou Y. Blockade of the C5a-C5aR axis alleviates lung damage in hDPP4-transgenic mice infected with MERS-CoV. *Emerg Microbes Infect*. 2018;7:77.

17. Sun S, Zhao G, Liu C, Fan W, Zhou X, Zeng L, Guo Y, Kou Z, Yu H, Li J, Wang R, Li Y, Schneider C, Habel M, Riedemann NC, Du L, Jiang S, Guo R and Zhou Y. Treatment with anti-C5a antibody improves the outcome of H7N9 virus infection in African green monkeys. *Clin Infect Dis*. 2015;60:586-95.

18. Sun S, Zhao G, Liu C, Wu X, Guo Y, Yu H, Song H, Du L, Jiang S, Guo R, Tomlinson S and Zhou Y. Inhibition of complement activation alleviates acute lung injury induced by highly pathogenic avian influenza H5N1 virus infection. *American journal of respiratory cell and molecular biology*. 2013;49:221-30.

19. Gao T, Hu M and Zhang X. Highly pathogenic coronavirus N protein aggravates lung injury by

MASP-2-mediated complement over-activation. https://doi.org/10.1101/2020.03.29.20041962. 2020.

20. Heja D, Kocsis A, Dobo J, Szilagyi K, Szasz R, Zavodszky P, Pal G and Gal P. Revised mechanism of complement lectin-pathway activation revealing the role of serine protease MASP-1 as the exclusive activator of MASP-2. *Proceedings of the National Academy of Sciences of the United States of America*. 2012;109:10498-503.

21. Zhou Y, Lu K, Pfefferle S, Bertram S, Glowacka I, Drosten C, Pohlmann S and Simmons G. A single asparagine-linked glycosylation site of the severe acute respiratory syndrome coronavirus spike glycoprotein facilitates inhibition by mannose-binding lectin through multiple mechanisms. *J Virol*. 2010;84:8753-64.

22. Magro C, Mulvey JJ, Berlin D, Nuovo G, Salvatore S, Harp J, Baxter-Stoltzfus A and Laurence J. Complement associated microvascular injury and thrombosis in the pathogenesis of severe COVID-19 infection: A report of five cases. *Transl Res*. 2020.

23. Sodhi CP, Wohlford-Lenane C, Yamaguchi Y, Prindle T, Fulton WB, Wang S, McCray PB, Jr., Chappell M, Hackam DJ and Jia H. Attenuation of pulmonary ACE2 activity impairs inactivation of des-Arg(9) bradykinin/BKB1R axis and facilitates LPS-induced neutrophil infiltration. *American journal of physiology Lung cellular and molecular physiology*. 2018;314:L17-L31.

24. Guy HR, Durell SR, Schoch C and Blumenthal R. Analyzing the fusion process of influenza hemagglutinin by mutagenesis and molecular modeling. *Biophys J*. 1992;62:95-7.

25. Kuba K, Imai Y, Rao S, Gao H, Guo F, Guan B, Huan Y, Yang P, Zhang Y, Deng W, Bao L, Zhang B, Liu G, Wang Z, Chappell M, Liu Y, Zheng D, Leibbrandt A, Wada T, Slutsky AS, Liu D, Qin C, Jiang C and Penninger JM. A crucial role of angiotensin converting enzyme 2 (ACE2) in SARS coronavirus-induced lung injury. *Nat Med*. 2005;11:875-9.

26. Debreczeni ML, Nemeth Z, Kajdacsi E, Schwaner E, Mako V, Masszi A, Doleschall Z, Rigo J, Walter FR, Deli MA, Pal G, Dobo J, Gal P and Cervenak L. MASP-1 Increases Endothelial Permeability. *Frontiers in immunology*. 2019;10:991.

27. Dobo J, Major B, Kekesi KA, Szabo I, Megyeri M, Hajela K, Juhasz G, Zavodszky P and Gal P. Cleavage of kininogen and subsequent bradykinin release by the complement component: mannose-binding lectin-associated serine protease (MASP)-1. *PLoS One*. 2011;6:e20036.

28. Kaplan AP and Ghebrehiwet B. The plasma bradykinin-forming pathways and its interrelationships with complement. *Mol Immunol*. 2010;47:2161-9.

29. Menter T, Haslbauer JD, Nienhold R, Savic S, Hopfer H, Deigendesch N, Frank S, Turek D, Willi N, Pargger H, Bassetti S, Leuppi JD, Cathomas G, Tolnay M, Mertz KD and Tzankov A. Post-mortem examination of COVID19 patients reveals diffuse alveolar damage with severe capillary congestion and variegated findings of lungs and other organs suggesting vascular dysfunction. *Histopathology*. 2020.

30. Noris M, Benigni A and Remuzzi G. The case of Complement activation in COVID-19 multiorgan impact. *Kidney Int*. 2020.

31. Nielsen EW, Waage C, Fure H, Brekke OL, Sfyroera G, Lambris JD and Mollnes TE. Effect of supraphysiologic levels of C1-inhibitor on the classical, lectin and alternative pathways of complement. *Molecular immunology*. 2007;44:1819-26.

32. Carugati A, Pappalardo E, Zingale LC and Cicardi M. C1-inhibitor deficiency and angioedema. *Molecular immunology*. 2001;38:161-73.

33. Hansen CB, Csuka D, Munthe-Fog L, Varga L, Farkas H, Hansen KM, Koch C, Skjodt K, Garred P and Skjoedt MO. The Levels of the Lectin Pathway Serine Protease MASP-1 and Its Complex Formation with C1 Inhibitor Are Linked to the Severity of Hereditary Angioedema. *Journal of immunology (Baltimore, Md : 1950)*. 2015;195:3596-604.

34. Davis B and Bernstein JA. Conestat alfa for the treatment of angioedema attacks. *Therapeutics and clinical risk management*. 2011;7:265-73.

35. van Veen HA, Koiter J, Vogelezang CJ, van Wessel N, van Dam T, Velterop I, van Houdt K, Kupers L, Horbach D, Salaheddine M, Nuijens JH and Mannesse ML. Characterization of recombinant human C1 inhibitor secreted in milk of transgenic rabbits. *J Biotechnol*. 2012;162:319-26.

36. Gesuete R, Storini C, Fantin A, Stravalaci M, Zanier ER, Orsini F, Vietsch H, Mannesse ML, Ziere B, Gobbi M and De Simoni MG. Recombinant C1 inhibitor in brain ischemic injury. *Annals of neurology*. 2009;66:332-42.

37. Igonin AA, Protsenko DN, Galstyan GM, Vlasenko AV, Khachatryan NN, Nekhaev IV, Shlyapnikov SA, Lazareva NB and Herscu P. C1-esterase inhibitor infusion increases survival rates for patients with sepsis*. *Critical care medicine*. 2012;40:770-7.

38. Caliezi C, Zeerleder S, Redondo M, Regli B, Rothen HU, Zurcher-Zenklusen R, Rieben R, Devay J, Hack CE, Lammle B and Wuillemin WA. C1-inhibitor in patients with severe sepsis and septic shock: beneficial effect on renal dysfunction. *Critical care medicine*. 2002;30:1722-8.

39. Hack CE, Ogilvie AC, Eisele B, Eerenberg AJ, Wagstaff J and Thijs LG. C1-inhibitor substitution therapy in septic shock and in the vascular leak syndrome induced by high doses of interleukin-2. *Intensive Care Med*. 1993;19 Suppl 1:S19-28.

40. Nurnberger W, Heying R, Burdach S and Gobel U. C1 esterase inhibitor concentrate for capillary leakage syndrome following bone marrow transplantation. *Ann Hematol*. 1997;75:95-101.

41. Castellano G, Melchiorre R, Loverre A, Ditonno P, Montinaro V, Rossini M, Divella C, Battaglia M, Lucarelli G, Annunziata G, Palazzo S, Selvaggi FP, Staffieri F, Crovace A, Daha MR, Mannesse M, van Wetering S, Paolo Schena F and Grandaliano G. Therapeutic targeting of classical and lectin pathways of complement protects from ischemia-reperfusion-induced renal damage. *Am J Pathol*. 2010;176:1648-59.

42. Panagiotou A, Trendelenburg M, Heijnen I, Moser S, Bonati LH, Breidthardt T, Fahrni G, Kaiser C, Jeger R and Osthoff M. A Randomized Trial of Recombinant Human C1-Esterase-Inhibitor in the Prevention of Contrast-Induced Kidney Injury. *JACC Cardiovasc Interv*. 2020.

43. Lu R, Zhao X, Li J, Niu P, Yang B, Wu H, Wang W, Song H, Huang B, Zhu N, Bi Y, Ma X, Zhan F, Wang L, Hu T, Zhou H, Hu Z, Zhou W, Zhao L, Chen J, Meng Y, Wang J, Lin Y, Yuan J, Xie Z, Ma J, Liu WJ, Wang D, Xu W, Holmes EC, Gao GF, Wu G, Chen W, Shi W and Tan W. Genomic characterisation and epidemiology of 2019 novel coronavirus: implications for virus origins and receptor binding. *Lancet*. 2020;395:565-574.

44. Jansen PM, Eisele B, de Jong IW, Chang A, Delvos U, Taylor FB, Jr. and Hack CE. Effect of C1 inhibitor on inflammatory and physiologic response patterns in primates suffering from lethal septic shock. *J Immunol*. 1998;160:475-84.

45. Dalle Lucca JJ, Li Y, Simovic M, Pusateri AE, Falabella M, Dubick MA and Tsokos GC. Effects of C1 inhibitor on tissue damage in a porcine model of controlled hemorrhage. *Shock*. 2012;38:82-91.

46. Zeerleder S, Caliezi C, van Mierlo G, Eerenberg-Belmer A, Sulzer I, Hack CE and Wuillemin WA. Administration of C1 inhibitor reduces neutrophil activation in patients with sepsis. *Clin Diagn Lab Immunol*. 2003;10:529-35.

47. Nurnberger W, Petrik K, Burdach S and Gobel U. C1 esterase inhibitor (C1-INH) can reduce plasma concentrations of the complement activation product C5a. *Intensive Care Med*. 1994;20:242.

48. Farrell C, Hayes S, Relan A, van Amersfoort ES, Pijpstra R and Hack CE. Population pharmacokinetics of recombinant human C1 inhibitor in patients with hereditary angioedema. *British journal of clinical pharmacology*. 2013;76:897-907.

49. van Doorn MB, Burggraaf J, van Dam T, Eerenberg A, Levi M, Hack CE, Schoemaker RC, Cohen AF and Nuijens J. A phase I study of recombinant human C1 inhibitor in asymptomatic patients with hereditary angioedema. *J Allergy Clin Immunol*. 2005;116:876-83.

50. Riedl MA, Grivcheva-Panovska V, Moldovan D, Baker J, Yang WH, Giannetti BM, Reshef A, Andrejevic S, Lockey RF, Hakl R, Kivity S, Harper JR, Relan A and Cicardi M. Recombinant human C1 esterase inhibitor for prophylaxis of hereditary angio-oedema: a phase 2, multicentre, randomised, double-blind, placebo-controlled crossover trial. *Lancet*. 2017;390:1595-1602.

51. Relan A, Bakhtiari K, van Amersfoort ES, Meijers JC and Hack CE. Recombinant C1-inhibitor: effects on coagulation and fibrinolysis in patients with hereditary angioedema. *BioDrugs*. 2012;26:43-52.

52. Nuijens JH, Eerenberg-Belmer AJ, Huijbregts CC, Schreuder WO, Felt-Bersma RJ, Abbink JJ, Thijs LG and Hack CE. Proteolytic inactivation of plasma C1- inhibitor in sepsis. *The Journal of clinical investigation*. 1989;84:443-50.

53. de Zwaan C, Kleine AH, Diris JH, Glatz JF, Wellens HJ, Strengers PF, Tissing M, Hack CE, van Dieijen-Visser MP and Hermens WT. Continuous 48-h C1-inhibitor treatment, following reperfusion therapy, in patients with acute myocardial infarction. *Eur Heart J*. 2002;23:1670-7.

54. Fu Y, Cheng Y and Wu Y. Understanding SARS-CoV-2-Mediated Inflammatory Responses:

From Mechanisms to Potential Therapeutic Tools. *Virol Sin*. 2020.

Additional References

1. Declaration of Helsinki, Version October 2013, (http://www.wma.net/en/30publications/10policies/b3/index.html)
2. International Conference on Harmonization (ICH, 1996) E6 Guideline for Good Clinical Practice. (http://www.ich.org/fileadmin/Public_Web_Site/ICH_Products/Guidelines/Efficacy/E6/E6_R2__Step_4.pdf)
3. International Conference on Harmonization (ICH, 1997) E8 Guideline: General Considerations for Clinical Trials http://www.ich.org/fileadmin/Public_Web_Site/ICH_Products/Guidelines/Efficacy/E8/Step4/E8_Guideline.pdf)
4. Humanforschungsgesetz, HFG Bundesgesetz über die Forschung am Menschen (Bundesgesetz über die Forschung am Menschen, HFG) vom 30. September 2011/ Loi fédérale relative à la recherche sur l'être humain (loi relative à la recherche sur l'être humain, LRH) du 30 septembre 2011 / Legge federale concernente la ricerca sull'essere umano (Legge sulla ricerca umana, LRUm) del 30 settembre 2011
5. Verordnung über klinische Versuche in der Humanforschung (Verordnung über klinische Versuche, KlinV) vom 20. September 2013 / Ordonnance sur les essais cliniques dans le cadre de la recherche sur l'être humain (Ordonnance sur les essais cliniques, OClin) du 20 septembre 2013. Ordinanza sulle sperimentazioni cliniche nella ricerca umana (Ordinanza sulle sperimentazioni cliniche, OSRUm) del 20 settembre 2013
6. Heilmittelgesetz, HMG Bundesgesetz über Arzneimittel und Medizinprodukte (Heilmittelgesetz, HMG) vom 15. Dezember 2000 / Loi fédérale sur les médicaments et les dispositifs médicaux (Loi sur les produits thérapeutiques, LPT) du 15 décembre 2000 / Legge federale sui medicamenti e i dispositivi medici (Legge sugli agenti terapeutici, LATer)
7. MEDDEV 2.7/3 revision 3, May 2015
8. Medizinprodukteverordnung (MepV) vom 17. Oktober 2001 / Ordonnance sur les dispositifs médicaux (ODim) du 17 octobre 2001 / Ordinanza relativa ai dispositivi medici (ODmed) del 17 ottobre 2001
9. WHO, International Clinical Trials Registry Platform (ICTRP) (http://www.who.int/ictrp/en/)
